# A Late Cretaceous diversification of Asian oviraptorid dinosaurs: evidence from a new species preserved in an unusual posture

**DOI:** 10.1038/srep35780

**Published:** 2016-11-10

**Authors:** Junchang Lü, Rongjun Chen, Stephen L. Brusatte, Yangxiao Zhu, Caizhi Shen

**Affiliations:** 1 Institute of Geology, Chinese Academy of Geological Sciences, Beijing 100037, China; 2 Dongyang Museum, Dongyang City 322100, Zhejiang Province, China; 3 School of GeoSciences, University of Edinburgh, Grant Institute, James Hutton Road, Edinburgh EH9 3FE, United Kingdom

## Abstract

Oviraptorosaurs are a bizarre group of bird-like theropod dinosaurs, the derived forms of which have shortened, toothless skulls, and which diverged from close relatives by developing peculiar feeding adaptations. Although once among the most mysterious of dinosaurs, oviraptorosaurs are becoming better understood with the discovery of many new fossils in Asia and North America. The Ganzhou area of southern China is emerging as a hotspot of oviraptorosaur discoveries, as over the past half decade five new monotypic genera have been found in the latest Cretaceous (Maastrichtian) deposits of this region. We here report a sixth diagnostic oviraptorosaur from Ganzhou, *Tongtianlong limosus* gen. et sp. nov., represented by a remarkably well-preserved specimen in an unusual splayed-limb and raised-head posture. *Tongtianlong* is a derived oviraptorid oviraptorosaur, differentiated from other species by its unique dome-like skull roof, highly convex premaxilla, and other features of the skull. The large number of oviraptorosaurs from Ganzhou, which often differ in cranial morphologies related to feeding, document an evolutionary radiation of these dinosaurs during the very latest Cretaceous of Asia, which helped establish one of the last diverse dinosaur faunas before the end-Cretaceous extinction.

Oviraptorosaurs are some of the most unusual dinosaurs. These bird-like, feathered theropods diverged dramatically from their close cousins, evolving shortened toothless skulls with a staggering diversity of pneumatic cranial crests in derived forms^1^. Unlike the stereotypical view of theropods as stealthy carnivores, oviraptorosaurs probably had more varied diets. Previous studies have suggested that oviraptorosaurs may have eaten such foods as eggs, mollusks, plants, shellfish, and nuts^1^,^2^,^3^,^4^,^5^,^6^,^7^,^8^,^9^,^10^,^11^, but these hypotheses remain to be more conclusively tested. The bizarre body plan and diets of oviraptorosaurs were clearly successful, as these dinosaurs were highly diverse in the Cretaceous of Asia and North America, where they ranged from the size of a turkey to nearly the length of 7 meters^12^. These theropods, once so poorly understood, are now recognized as important components of terrestrial food webs in the northern continents during the final ~15 million years of the Age of Dinosaurs^13^.

Our understanding of oviraptorosaur anatomy and evolution has greatly increased with a bounty of new discoveries over the past decade. In total, more than 35 oviraptorosaur genera are now known. Many of the recent discoveries have come from China, particularly from three areas of the country: northern China (including Inner Mongolia and western Liaoning Province^8^,^12^,^14^,^15^,^16^,^17^,^18^,^19^,^20^), central China (Henan Province^21^,^10^), and southern China (including Guangdong and Jiangxi provinces^9^,^22^,^23^,^24^,^25^,^26^,^27^,^28^,^29^,^30^). The southern Chinese oviraptorosaurs are especially diverse. Over the last five years alone, five distinct taxa have been described from the Ganzhou area of Jiangxi Province, all of which are highly derived, toothless oviraptorids represented, thus far, by a single holotype individual. Because of these discoveries, the latest Cretaceous (Maastrichtian) deposits of the Ganzhou area becoming critical for understanding the evolution of this aberrant dinosaur subgroup.

We here report a sixth diagnostic oviraptorosaur from the Ganzhou area (Fig. 1), represented by a remarkably well-preserved specimen in an unusual posture with its limbs splayed to the side, its neck outstretched, and its head raised. This specimen is described as a new species, *Tongtianlong limosus* gen. et sp. nov., based on its unique dome-like skull roof, highly convex premaxilla, and many additional features setting it apart from other oviraptorosaurs, both from Ganzhou and globally. The discovery of yet another diagnostic specimen from Ganzhou begs the question of why so many oviraptorosaurs are found here. We argue that the high variability of oviraptorosaurs documents an evolutionary radiation of these dinosaurs in the very latest Cretaceous of Asia, perhaps enabled by differences in skull morphology related to feeding. This flurry of evolution helped establish some of the final dinosaur faunas before the end-Cretaceous extinction.

## Results

### Systematic Paleontology

Dinosauria Owen, 1842^31^.

Theropoda Marsh, 1881^32^.

Maniraptora Gauthier, 1986^33^.

Oviraptorosauria Barsbold, 1976^34^.

Oviraptoridae Barsbold, 1976^34^.

**Figure 2:**
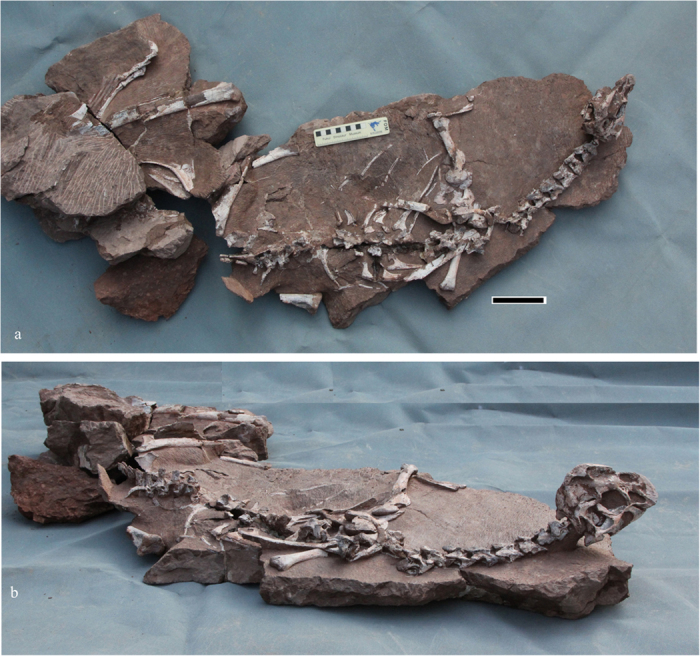
The whole skeleton of the holotype *Tongtianlong limosus* gen. et sp. nov. in dorsal view (**a**) and lateral view (**b**). Scale bar = 10 cm.

**Figure 3:**
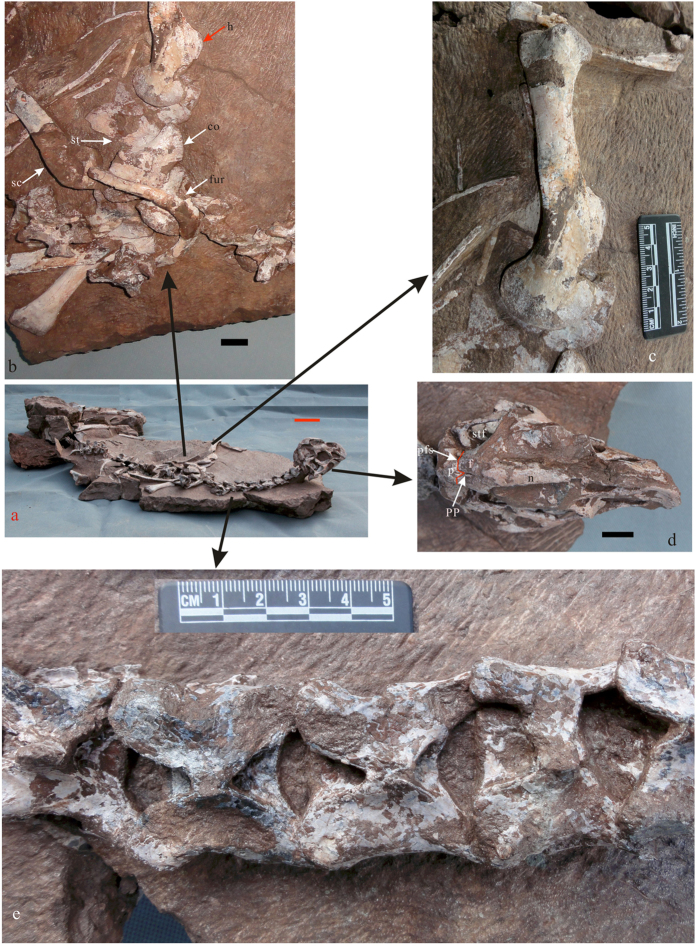
The holotype of *Tongtianlong limosus* gen. et sp. nov.: whole skeleton (**a**), close-up of furcula (**b**), close up of humerus (**c**), dorsal view of skull (**d**) and dorsal view of middle cervical vertebrae. Scale bars = 10 cm in (**a**) and 2 cm in (**b**,**d**); Abbreviation: co, coracoids; f, frontal; fur, furcula; h, humerus; p, parietal; pfs, frontal/parietal suture; pp, parietal process; sc, scapula; st, sternum; stf, supratemporal fenestra.

**Figure 4:**
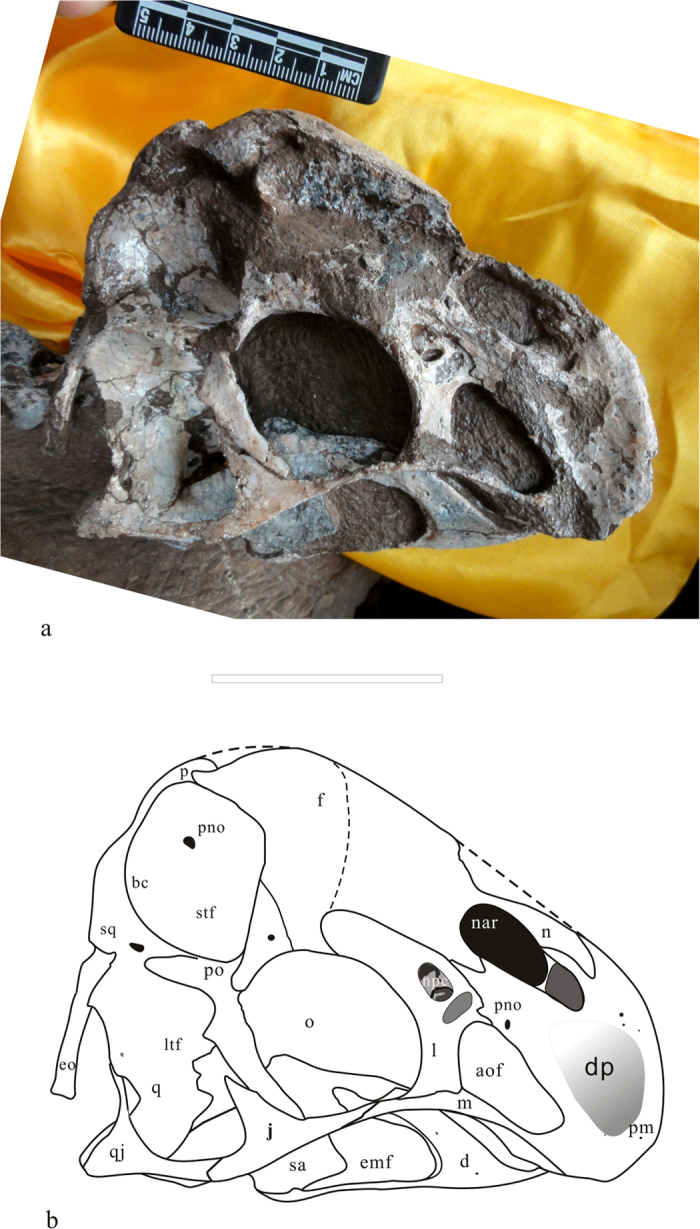
The photograph (**a**) and line drawing (**b**) of the skull: *Tongtianlong limosus* gen. et sp. nov. in right lateral view. Abbreviations: aof, antorbital fenestra; bc, braincase; d, dentary; emf, external mandibular fenestra; eo, exoccipital; f, frontal; j, jugal; l, lacrimal; ltf: lower temporal fenestra; m, maxilla; n, nasal;nar, narial opening; npc, nasopharyngeal canal; o, orbit; p, parietal; pm, premaxilla; pno, pneumatic opening; po, postorbital;q, quadrate;qj, quadratojugal; sa, surangular; sq, squamosal; stf, supratemporal fenestra. Scale bar = 5 cm.

*Tongtianlong limosus* gen. et sp. nov. (Figs 2, 3 and 4)

### Etymology

**Figure 5:**
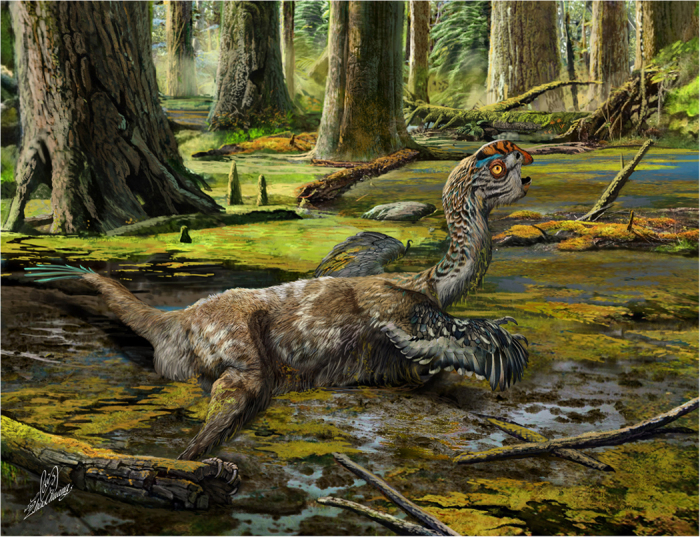
An artistic reconstruction, showing the last-ditch struggle of *Tongtianlong limosus* as it was mired in mud, one possible, but highly speculative, interpretation for how the specimen was killed and buried (Drawn by Zhao Chuang).

Tongtian, Chinese Pinyin, refers to Tongtianyan of Ganzhou, the first grotto south of the Yangtze River. Tongtian also means the road to heaven, a fitting epitaph for a deceased dinosaur preserved with outstretched arms. Long, Chinese Pinyin for dragon. Limosus, Latin for muddy, refers to the holotype specimen being found in an unusual posture in a mudstone (Fig. 5).

### Holotype

A nearly complete, three-dimensionally preserved skeleton with skull and lower jaws (DYM-2013-8). The specimen is accessioned at the Dongyang Museum, Dongyang City, Zhejiang Province.

### Type locality and horizon

The building site of the No. 3 high school of Ganxian (GPS coordinates are provided on request from the first author); Nanxiong Formation (Maastrichtian, Upper Cretaceous)^35^.

### Diagnosis

Oviraptorid dinosaur with the following unique combination of characters, with autapomorphies among all oviraptorosaurs indicated with an asterisk and autapomorphies among oviraptorids indicated with a double asterisk (these latter features are present in some caenagnathids): dome-like skull roof with highest point located above the posterodorsal corner of the orbit*; anterior margin of the premaxilla highly convex in lateral view*; distinct process at the middle of the anterior margin of the parietal on the skull roof*; plate-like lacrimal shaft that is anteroposteriorly long in lateral view, with a flat lateral surface*; foramen magnum smaller than the occipital condyle**; absence of symphyseal ventral process of the dentary**; absence of distinct lateral xiphoid process of the sternum posterior to the costal margin**.

*Tongtianlong* differs from other Ganzhou oviraptorids with preserved skull material (*Banji*, *Huanansaurus*) in that the anteroventral corner of the external naris is far above a horizontal line tangent with the posterodorsal corner of the antorbital fenestra, an unusual feature otherwise only seen in *Nemegtomaia*^36^,^37^,^38^ and *Rinchenia* (Barsbold^39^) (=*Oviraptor mongoliensis*^1^,^40^). *Tongtianlong* also differs from other Ganzhou oviraptorids in numerous ways that are encapsulated in the character scores in our phylogenetic analysis. *Tongtianlong* differs from *Banji*^26^ in possessing a postorbital process of the jugal that is posterodorsally inclined relative to the ventral ramus (not perpendicular), lacking a downturned symphyseal portion of the dentary (see also Supplementary Information Fig. S1a), lacking a prominent process on the posteroventral surface of the dentary symphysis, and possessing a more anteroposteriorly elongate external mandibular fenestra.

*Tongtianlong* is distinguished from *Ganzhousaurus*^27^ in lacking a downturned symphyseal portion of the dentary, possessing a more anteroposteriorly elongate external mandibular fenestra, having a dentary that contributes to the ventral border of the external mandibular fenestra (not excluded from the border by the anterior extension of the angular), lacking a depression on the lateral surface of the dentary immediately anterior to the external mandibular fenestra, and possessing a metatarsal III that is anteroposteriorly flattened with a concave posterior surface (not ovoid or subtriangular in cross section).

*Tongtianlong* is different from *Jiangxisaurus*^28^ in lacking a downturned symphyseal portion of the dentary. Furthermore, the ratio of radius length to humerus length (78%) and the height-to-length ratio of the lower jaw (34%) are greater than those of *Jiangxisaurus* (70% and 20%, respectively).

*Tongtianlong* differs from *Nankangia*^9^ in lacking a prominent process on the posteroventral surface of the dentary symphysis, lacking a deep fossa on the lateral surface of the dentary, having a dentary that contributes to the dorsal border of the external mandibular fenestra (not excluded from the border by the anterior extension of the surangular), and lacking a depression on the lateral surface of the dentary immediately anterior to the external mandibular fenestra.

**Figure 6:**
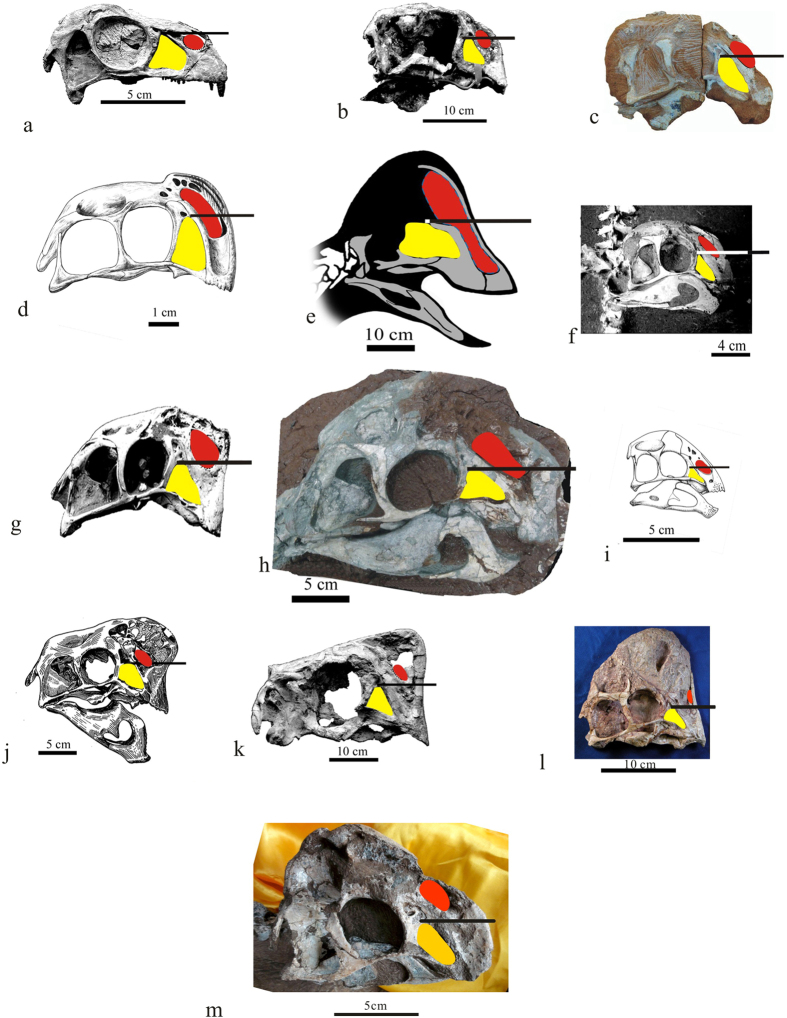
Skull comparisons of oviraptorosaurs showing relative positions of the posterodorsal corner of the antorbital fenestra and the anteroventral corner of the external narial opening. (**a**) *Incisivosaurus gauthieri*; (**b**) *Conchoraptor gracilis*; (**c**) *Wulatelong gobiensis*; (no scale) (**d**) *Banji long*; (**e**) *Anzu wyliei*; (**f**) *Khaan mckennai*; (**g**) *Citipati osmolskae*; (no scale) (**h**) *Huanansaurus ganzhouensis* (reversed); (**i**) *Yulong mini*; (**j**) *Oviraptor philoceratops*; (**k**) *Nemegtomaia barsboldi*; l: “*Oviraptor*” *mongoliensis*; m: *Tongtianlong limosus* gen. et sp. nov. (**a**,**b**,**f**,**g**,**j**,**k**) and l are from Lü^23^; (**c**) is modified from Xu *et al*.^18^; (**d**) is modified from Xu and Han^26^; (**e**) is modified from Lamanna *et al*.^41^ (reversed), (**i**) is from Lü *et al*.^10^ and (**h**) is from Lü *et al*.^29^. External narial opening is in red, and antorbital fenenstra is in yellow. Note: The horizontal line projected through the posterodorsal corner of the antorbital fenestra is parallel to the line linking the articular end of the quadrate and the ventral margin of the premaxilla. Modified from Lü *et al*.^29^.

*Tongtianlong* differs from *Huanansaurus*^29^ in skull morphology and forelimb proportions. There is a crest in *Huanansaurus* but not in *Tongtianlong*. The dorsal margin of the lower jaw, from the anterior tip to the coronoid eminence, is smoothly convex in *Tongtianlong*, whilst it is wave-like in *Huanansaurus*. The antorbital fenestra is sub-oval in *Tongtianlong*, but triangular in *Huanansaurus*. The ratio of radius length to humerus length in *Tongtianlong* (0.78) is much smaller than that of *Huanansaurus* (0.97). The anteroventral corner of the external naris is slightly below the horizontal line projected through the posterodorsal corner of the antorbital fenestra in *Huanansaurus*, whilst it is far above this line in *Tongtianlong* (Fig. 6).

## Description

The specimen is very well preserved in three dimensions, with the bones in natural articulation (Figs 2 and 3a; see also Supplementary Information Table S1). The limbs are splayed out sideways relative to the trunk, and the neck is curved upwards, such that the head is elevated relative to the remainder of the body. Because the specimen was collected by a farmer and construction workers, and it was not mapped *in situ* while being excavated, it is difficult to interpret what biological and/or taphonomic processes caused this strange posture. Judging by the fine state of preservation, the specimen probably was originally complete or nearly complete. However, some portions of the skeleton are missing, such as the distal regions of the arms, the right pelvic girdle and hind leg, and parts of the tail. This is because the specimen was collected by workers at an active construction site. The specimen was exposed after workmen blasted away some of the surrounding rocks with TNT; a drill hole where TNT was placed can be seen near the pelvic girdle.

The skull is almost completely preserved. It is missing only small portions of the anterior end of the premaxilla and nasals, and a small part of the right lower jaw. The most salient feature of the skull is that the cranial roof is dome-like, with its highest point above the posterodorsal corner of the orbit. Many other oviraptorosaurs possess cranial ornaments, which in some cases are elaborate and highly pneumatic (Fig. 4). However, in other taxa these crests are usually thinner (such as in *Nemegtomaia*^36^,^37^,^38^, it is 6 mm) than the dome-like condition in *Tongtianlong*. Furthermore, in other taxa these crests are peaked further anteriorly relative to *Tongtianlong*, either at the anterior end of the snout above the external naris and antorbital fenestra (as in *Banji*, *Citipati*, *Oviraptor*, and *Nemegtomaia*), or at approximately the midpoint of the cranium above the orbit (as in *Rinchenia*, *Huanansaurus*, and caenagnathids like *Anzu*^41^) (Fig. 6). Therefore, the posteriorly-peaked dome-like crest of *Tongtianlong* is autapomorphic among oviraptorosaurs, and a novel type of cranial ornamentation in this highly variable clade. The fine three-dimensional preservation of the specimen ensures that the shape of the dome-like crest is not an artefact of crushing or deformation.

There are five main openings in the cranium, as is standard for oviraptorosaurs and other dinosaurs. Anteriorly a large, oval-shaped external naris is positioned above a slightly smaller, triangular antorbital fenestra (Fig. 4). The anteroventral corner of the naris is located far above the level of the posterodorsal corner of the antorbital fenestra, which is also the case in *Nemegtomaia* and *Rinchenia*, but differs from the condition in most other oviraptorosaurs, in which the naris extends further ventrally so that it reaches past the posterodorsal corner of the antorbital fenestra (Fig. 6). The orbit is large and nearly circular, as is typical for oviraptorosaurs. The lateral temporal fenestra is the largest opening in the skull. It is rectangular, with a long axis that extends slightly anteroventrally, which differs from the more circular or square fenestrae of many other oviraptorosaurs. The supratemporal fenestra is positioned above the lateral temporal fenestra and is partially visible in lateral view. It is much smaller than the orbit and lateral temporal fenestra.

The premaxilla is toothless like in all derived oviraptorosaurs. The left and right premaxillae appear to be unfused to each other, based on open sutures in the region of the broken dorsal surfaces of both bones, but the premaxilla and maxilla are fused together without a clear sutural trace. The anterior margin of the premaxilla is highly convex, which is an autapomorphy of *Tongtianlong*. Most other oviraptorosaurs have a straight anterior premaxilla (e.g., *Citipati*^42^, *Khaan*^42^), and this is also the case in the Ganzhou oviraptorid *Huanasaurus*^29^. *Yulong* and the Ganzhou oviraptorid *Banji* have a slightly rounded anterior margin of the premaxilla in lateral view, but it is not nearly as convex as in *Tongtianlong*. The premaxilla is divided into two branches, both of which extend posterodorsally. The upper one forms the anterodorsal margin of the external naris, whereas the much wider lower one forms most of the anterodorsal margin of the antorbital fenestra, thus separating the naris from the antorbital fenestra and completely excluding the maxilla from the narial border. The posterior end of this branch overlaps the lateral surface of the lacrimal. The divergence of the two branches defines the shape of the external naris. Anteroventral to the naris, a deeply concave fossa extends on the lateral surface of the premaxilla, as in *Huanasaurus*^29^, *Yulong*^10^, and *Nemegtomaia*^37^,^38^, but unlike the slightly concave surface in *Citipati*^6^. The maxilla is very small and exposed only as a tiny sliver of bone in lateral view. It forms the ventral margin of the antorbital opening and lacks teeth, but has a small triangular ‘tooth-like’ process on its ventral surface.

The lacrimal is divided into three branches: a short anterior process that is covered by the premaxilla, a bulbous posterior process that extends dorsally to define the anterodorsal corner of the orbit, and a large ventral shaft. The shape of the shaft is unique: whereas in other oviraptorosaurs the lacrimal shaft is gracile (thin anteroposteriorly) and has at least a partially convex lateral surface (Fig. 6), in *Tongtianlong* it is robust (thick anteroposteriorly) with a flat lateral surface (Fig. 4). In effect, the lacrimal shaft of *Tongtianlong* is plate-like, which is considered an autapomorphy of the taxon. In the region where the three processes meet, the lateral surface of the lacrimal is penetrated by a large opening (called the nasopharyngeal canal by Balanoff and Norell^43^) that leads into an internal recess, which is further subdivided internally. Anteroventral to this pneumatic opening is an ovoid fossa on the lateral surface of the lacrimal, which is probably also pneumatic in origin, and which may also invade the bone internally, although poor preservation makes this difficult to confirm. Complex pneumaticity in this region is common in oviraptorosaurs^6^,^43^). However, the pneumatic openings in *Tongtianlong* are much larger and more elaborate than the corresponding pneumaticity in the two Ganzhou oviraptorids with well-preserved cranial material, *Huanansaurus*^29^ and *Banji*^26^.

The postorbital is triradiate, with short anterior and posterior processes and a very long ventral process that projects anteroventrally, terminating at the floor of the orbit. The slender and elongate jugal is divided into three branches. The rod-like anterior process contacts the lacrimal and maxilla. The short ascending process extends posterodorsally to make up approximately half of the postorbital bar separating the orbit and lateral temporal fenestra. The posterior process contacts the quadratojugal underneath the lateral temporal fenestra. Here, the jugal overlaps the quadratojugal laterally, and the two bones are sutured but not fused. The quadratojugal is tightly appressed to the lateral surface of the quadrate, and it does not appear that the two bones could move relative to each other. There is, however, a small fenestra between the small dorsal process of the quadratojugal and the lateral margin of the quadrate. The dorsal part of quadrate is bent backwards, and there is an opening on the anterior surface of the quadrate which indicates that the bone is pneumatized.

On the skull roof, the dorsal surface of the posterior portion of the nasal is smoothly convex. The left and right nasals are fused, without any sign of a suture between them. The lateral surface of the nasal is strongly concave, and although the surface is not well preserved, visible regions of original bone texture indicate extreme pneumaticity in this region, as is standard for derived oviraptorosaurs. The nasal-frontal suture is V-shaped in dorsal view. The frontals are short anteroposteriorly, and the left and right elements are not fused on the midline. The two parietals are fused to each other, but not to the frontals and there is no parietal crest (see also Supplementary Information, Fig. S1b). The frontal-parietal suture is mostly straight, but there is a distinct process extending forwards from the middle of the anterior margin of the parietal (Fig. 3d, pp). This process is wedged between the frontals. It is considered an autapomorphy of *Tongtianlong*, as it is absent in other oviraptorosaurs.

Portions of the braincase are visible in lateral and posterior view. The supraoccipital is triangular, which a concave posterior surface. The exoccipital-opisthotic forms the dorsal margin of the foramen magnum, thus separating the supraoccipital from the foramen margin. The exoccipital tapers as it extends lateroventrally. The occipital condyle is larger than the foramen magnum, a condition that is seen in some caenagnathids (e.g., *Anzu*^41^), but differs from the proportionally smaller occipital condyles of other oviraptorids. The occipital condyle is located posterior to the articular condyles of the quadrate.

The mandible is nearly complete. In lateral view, the ventral margin of the lower jaw is straight. The anterior end of the dentary is not as strongly downturned as in other derived oviraptorids. There is no depressed fossa on the lateral surface of the dentary immediately anterior to the external mandibular fenestra, and there are no articular grooves for the dentary on the ventrolateral edge of the angular and the dorsal surface of the surangular. The dentary contributes widely to the dorsal and ventral margins of the external mandibular fenestra, which is more anteroposteriorly elongated than the circular fenestrae of many other oviraptorids. The posterior part of the surangular is strongly concave laterally, and is pierced by a small opening.

Postcranially, the neck is comprised of 11 cervical vertebrae. The first nine of these are preserved in natural articulation, with their dorsal surfaces exposed. In dorsal view the anterior-middle cervicals are roughly square shaped, as defined by lines drawn between the posterior margins of the postzygapophyses, the anterior margins of the prezygapophyses, and the lateral edge of the vertebra (Fig. 3e). They become more rectangular in shape, longer than wide, more posteriorly in the neck. The neural spines are very small, as they are reduced to tiny peg-like projections at the center of the neural arches. The epipophyses are well developed in the second, third and fourth cervical vertebrae, but they become smaller in the middle cervicals and then disappear posterior to the sixth vertebra. There is a pneumatic opening (=pleurocoel) visible on the slightly exposed lateral centrum surface of the second cervical, but the lateral surfaces of the remaining cervicals are covered by matrix. The isolated posterior cervical vertebra, which is not in close articulation with the rest of the neck and therefore more widely exposed than the others, has a concave anterior articular surface of the centrum and a slightly convex posterior articular surface.

The dorsal vertebrae were heavily damaged during collection, so few details of their morphology can be observed. The neural spines of the posterior dorsals are tall and slightly expanded anteroposteriorly. Some dorsal ribs are present on both sides of the specimen, none of which exhibit any pneumatic openings on their proximal ends. The sacrum is not well preserved, but the anterior neural spines are clearly unfused to each other and were closely appressed to the medial surface of the ilium in dorsal view. There appears to be a pneumatic foramen (=pleurocoel) on the final sacral vertebra, and the lateral ends of the fused transverse processes and sacral ribs are strongly expanded anteroposteriorly, with rounded dorsal surfaces. Part of the distal tail is missing, but there are at least 19 caudal vertebrae. The caudals are rectangular in dorsal view, with elongate transverse processes that extend laterally and slightly posteriorly. One laterally exposed anterior caudal has a small opening that appears to be pneumatic in nature. The haemal arches are very long.

Portions of the shoulder girdles and proximal forearms are present on both sides of the specimen. The scapula is slender and curved medially. Its proximal end is expanded but not fused to the coracoid, the two bones forming an angle of approximately 130 degrees when in articulation. The coracoid is quadrangular in shape and has a large distally tapering posteroventral process, which extends slightly past the glenoid and is rounded at its end. The lateral surface of the coracoid is convex, the distinct biceps tubercle is located anterior to the glenoid, and the small and elongated coracoid foramen is positioned between the dorsal margin of the bone and the biceps tubercle. The medial surface of the coracoid is deeply concave and the coracoid foramen is expressed as a much larger, more circular opening than on the lateral surface. The thin sternum is a single element consisting of fused left and right components. It lacks a lateral xiphoid process and there is no groove for the coracoids along its anterior margin. The furcula is a broadly U-shaped, with a short ventral process on the midline and flattened distal ends (Fig. 3b). The humerus has a long deltopectoral crest, which extends distally for nearly half the length of the shaft (Fig. 3c). The shaft is slightly twisted as in *Heyuannia*^22^ and *Nankangia*^9^. Part of the radius is preserved on the left side, but the ulnae and more distal forelimb elements are missing.

Very few details of the pelvic girdle are apparent, due to damage that occurred during collecting. Parts of the ilium and pubis are present but little can be said of their morphology, although the preserved portions indicate that the pelvis is mesopubic and the distal ends of the left and right pubes are not fused together. The ischia are better preserved on the left side. The posterior margin of the shaft is deeply concave, the distal margin of the obturator process is straight, and the lateral surface of the bone is concave. The tibia is longer than the femur. It has a straight shaft, a well-developed cnemial crest, and an expanded distal end with a concave posterior surface. The astragalus is tightly appressed to the distal tibia. In posterior view, the ventral margin of the astragalus is concave dorsally, and in anterior view the ascending process is taller than wide. Two flattened distal tarsals are fused to each other and the proximal metatarsals. Distal tarsal III, which covers the proximal ends of metatarsals II and III, is larger than distal tarsal IV, which covers the proximal end of metatarsal IV. The left pes is partially preserved (See also Supplementary Information Fig. S2). Metatarsal III is longer than metatarsal II, which is longer than metatarsal IV. Metatarsal III remains visible along the length of the metatarsaus, with only a slight constriction near its proximal end. Metatarsal V is short and rod-like with a pointed distal end. It is approximately 35% of the length of metatarsal V. The single visible pedal ungual is slightly curved.

### Phylogenetic analysis

*Tongtianlong* can clearly be assigned to Oviraptoridae based on numerous characters that are diagnostic of the clade (or proximal nodes within Oviraptorosauria), including: a pneumatic premaxilla; a medially inset subantorbital portion of the maxilla; fused nasals; a laterally projecting medial part of the lacrimal shaft that forms a flattened transverse bar in front of the eye; pneumatic skull roof bones; left and right iliac blades closely approaching or contacting each other on the midline^1^; and proximal caudals with pneumatized centra^44^.

We added *Tongtianlong* to a modified version of the phylogenetic dataset of Lü *et al*.^29^, which itself was an updated version of the dataset of Lamanna *et al*.^41^. We changed some characters that were previously multistate characters combining absence/presence and morphological differences into two separate characters, and also ordered multistate characters that describe a progressive sequence of size or morphological change. The data matrix now includes 43 taxa scored for 237 characters (see Methods and Supplementary Information).

**Figure 7:**
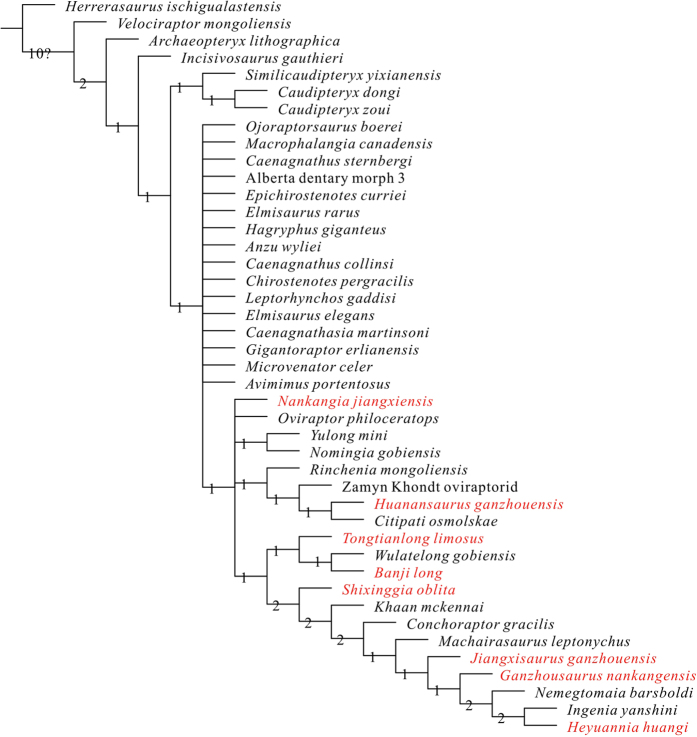
Strict consensus of 33104 most parsimonious trees obtained by TNT, based on analysis of 43 taxa and 237 characters, showing the phylogenetic position of *Tongtianlong limosus* gen. et sp. nov. (Tree length = 566, consistency index = 0.484 and retention index = 0.676). Numbers adjacent to each node are Bremer support values. All the oviraptorid dinosaurs from southern China are in red.

The strict consensus of the 33,104 most parsimonious trees recovers *Tongtianlong* as deeply nested within Oviraptoridae (synapomorphies for Oviraptoridae and other major clades largely follow previous analyses of this dataset, and won’t be repeated here) (Fig. 7). *Tongtianlong* is the sister taxon to a sister-taxon pair of the Ganzhou oviraptorid *Banji*^26^ and *Wulatelong* from the Campanian of Inner Mongolia^18^. The subclade comprised of these three taxa is united by three synapomorphies: the lack of a sagittal crest along the interparietal contact (character 30), a jugal process of the postorbital that extends far ventrally (character 36), and the presence of a surangular foramen (character 94). *Tongtianlong* is not recovered as a particularly close relative of any of the four other Ganzhou oviraptorids. Of these, *Nankangia* is placed within a polytomy as one of the most basal oviraptorids, *Huanansaurus* is recovered as an ‘intermediate’ grade oviraptorid that is outside of the clade consisting of *Tongtianlong* and more derived oviraptorids, and *Jiangxisaurus* and *Ganzhousaurus* are positioned as very highly nested oviraptorids, as successive outgroups to the specialized subclade centered on *Ingenia*.

The phylogenetic separation between *Tongtianlong* and other Ganzhou oviraptorids provides further evidence for their generic separation. It is not outside of the realm of possibility, however, that future work on oviraptorosaur ontogeny may show that *Tongtianlong* is synonymous with another Ganzhou taxon. If this is the case, we suggest that *Banji* would be the most likely con-specific, as it is the most closely related to *Tongtianlong* and is based on a much smaller holotype that conceivably could belong to a juvenile^26^. With that said, we consider the phylogenetic separation of *Tongtianlong* and *Banji*, the possession of numerous autapomorphies in *Tongtianlong* that are not seen in *Banji*, and the many character differences between the holotypes of *Tongtianlong* and *Banji* to be strong evidence that the two are distinct taxa, based on our current understanding of oviraptorosaur ontogeny and morphology.

## Discussion

*Tongtianlong* is the sixth oviraptorosaurian taxon named from the Nanxiong Formation of the Ganzhou area of Jiangxi Province, southern China. All of these have been described over the past five years, and include: *Banji*^26^, *Ganzhousaurus*^27^, *Jiangxisaurus*^28^, *Nankangia*^9^, and *Huanansaurus*^29^. Additionally, another oviraptorosaur taxon is known from the Nanxiong Formation in neighboring Guangdong Province, *Shixinggia*^24^. Because of these discoveries, this part of southern China has rapidly become one of the best areas in the world for oviraptorosaur fossils, and therefore a keystone area for understanding the evolution of this highly aberrant group of bird-like feathered dinosaurs.

The recent discoveries beg the question: why are so many oviraptorosaur taxa found in southern China? There are at least two possible explanations for the pattern, which are not mutually exclusive. First, it could be that the rush of recent discoveries has led to taxonomic over-inflation, and some of the specimens described as new species may be ontogenetic or sexually dimorphic forms of previously recognized species. Second, the Nanxiong Formation may be documenting a genuine radiation of oviraptorosaurs, an evolutionary event in which these small-to-mid-sized animals blossomed into many species and during the final few million years of the Age of Dinosaurs. Testing these two scenarios is currently difficult, but evidence is emerging that we argue favors the second scenario.

Regarding the first explanation, it may be that some of the six named Ganzhou oviraptorids are based on holotypes that actually belong to one of the other species. There is no doubt that the six oviraptorosaurs are anatomically distinct from each other, as each can be diagnosed by autapomorphies and/or a unique combination of characters. The new taxon *Tongtianlong*, for example, has four unique features that are not seen in any other oviraptorosaur, three additional features that are not seen in any other oviraptorid, and numerous differences with the other five Ganzhou taxa. However, not all morphological differences must be due to taxonomic separation. Differences between specimens could be caused by ontogeny, sexual dimorphism, or random variation. If the differences between the Ganzhou oviraptorids are not entirely taxonomic in nature, we suggest that the most likely culprit is ontogeny, particularly because some of the specimens differ in size (e.g., the *Banji* holotype is considerably smaller than the *Tongtianlong* holotype).

At the present time, all we can do is suggest that ontogenetic differences could potentially explain some of the variation seen in Ganzhou oviraptorids, but we cannot assess this with much certainty. Unfortunately, very little is known about how the anatomy of theropod dinosaurs changed during ontogeny, because most species are represented by very few fossils that fall far short of forming an ontogenetic sequence of hatchling to adult. The one exception is derived tyrannosaurids^45^,^46^, but these theropods are drastically different from oviraptorosaurs in their phylogenetic position, body sizes, diets, and ecological habits. A better parallel may be herbivorous dinosaurs like ceratopsids and hadrosauroids, which although distantly related to oviraptorosaurs were similar in possessing often gaudy head crests and other cranial ornamentation. It is well known that the ornamentation in these dinosaurs changed dramatically during ontogeny^47^,^48^,^49^,^50^,^51^,^52^. Therefore, we would expect the head ornaments of oviraptorosaurs to change during growth, making it potentially very difficult to distinguish ontogenetic morphs from separate species in the absence of histological data (which is currently unavailable for the Ganzhou oviraptorids because of the logistical difficulty of destructive sampling) or extremely large sample sizes.

We argue, however, that ontogeny probably does not explain most of the extreme variation seen among Ganzhou oviraptorids. Rather, we hypothesize that this variation is taxonomically informative. Although oviraptorosaur cranial ornaments probably did change during ontogeny, very few of the diagnostic characters of the Ganzhou oviraptorids are based on crest morphology. Most of the anatomical differences between species described as separate taxa concern the shapes and positions of cranial openings, the shapes and orientations of facial bones, and particularly features of the beak, lower jaw, and cranial muscle attachments that are likely related to feeding. For example, the new taxon *Tongtianlong* has a highly convex anterior premaxilla that is unique among oviraptorosaurs and differs from other Ganzhou species in presenting a prominent process on the posteroventral surface of the dentary symphysis, and a deep fossa on the lateral dentary and sagittal crest on the skull roof (both muscle attachment sites). Unless cranial musculature and feeding habits changed drastically during the lifetime of an individual oviraptorosaur, we currently hold that these differences among taxa are better explained by taxonomic separation (perhaps driven by feeding-related niche-partitioning, related to the peculiar but still poorly understood diets of oviraptorosaurs) rather than ontogeny. This hypothesis is bolstered by the recent discovery of very small ‘baby’ oviraptorids from central China, which already exhibit classic adult features of cranial fusion and deep lower jaws that are tied to large jaw muscles and strong bite forces^10^.

These arguments lead us to conclude that the great diversity of named oviraptorids from the Nanxiong Formation of southern China is genuine. In other words, there really was a variety of different oviraptorid species in this area during the latest Cretaceous. This is not unprecedented: one of the very few well-sampled small theropod faunas, the Yixian Formation of northeastern China, exhibits a staggering diversity of small carnivorous and omnivorous dromaeosaurids^53^,^54^,^55^,^56^. It may be that a high diversity of small theropods was common in individual dinosaur faunas, but has gone unrecognized because of preservational bias against small dinosaur fossils^57^,^58^.

With that said, it is also possible that many of the various Ganzhou oviraptorids did not actually live together. The Nanxiong Formation ranges from 600 to 7300 meters thick^35^, is very poorly dated, and its stratigraphy has not yet been studied in detail, making it difficult to determine the relative stratigraphic positions of different oviraptorid specimens. We are very much still in the initial exponential phase of collecting in the Ganzhou region: the wealth of new fossil discoveries over the past five years is the direct result of a burst of construction activity in the region. Many areas remain to be explored, many fossils remain to be collected, and much work on the local geology is clearly needed. It may turn out that the Nanxiong Formation spans a long length of time and/or that the individual oviraptorid specimens are widely separated from each other stratigraphically.

We suspect that the story of dinosaur evolution in the Nanxiong Formation may turn out to be similar to that in the Horseshoe Canyon and Dinosaur Park Formations of western Canada, two fossil-rich units that were also deposited in the latest Cretaceous. Initial exploration of these units in the early-mid 20^th^ century produced a fortune of dinosaur fossils, most notably numerous species of ceratopsids and hadrosauroids. As the geology of these formations became better understood and collecting was undertaken in a more rigorous manner, it became apparent that each formation spanned a few million years of time, and that the dinosaurs were not homogeneously distributed throughout the entire sequence^59^,^60^. Instead, unique and often short-lived assemblages of dinosaurs evolved, went extinct, and then were replaced by another assemblage. These formations record evolutionary radiations of dinosaurs: rapid evolution of many species, most likely enabled by dietary and ecological differences and possibly driven by environmental changes. We hypothesize that the Ganzhou oviraptorids underwent their own evolutionary radiation during the latest Cretaceous, in one of the final flurries of dinosaur evolution before the end-Cretaceous asteroid impact knocked out all non-avian species and ushered in the Age of Mammals.

## Methods

### Phylogenetic analysis

To determine the phylogenetic position of *Tongtianlong* within Oviraptorosauria, we added this taxon to a modified version the phylogenetic dataset of Lü *et al*.^29^. This is an updated version of the dataset of Lamanna *et al*.^41^, which includes a comprehensive sample of nearly all oviraptorosaurs scored for phylogenetically informative features of the skeleton. With the addition of *Tongtianlong*, the data matrix now includes 43 taxa (40 oviraptorosaurs plus *Herrerasaurus*, *Velociraptor*, and *Archaeopteyx* as outgroups) scored for 237 characters. We slightly modified some multistate characters and character ordering relative to Lamanna *et al*.^41^, as explained in the supplement.

We subjected the dataset to a maximum parsimony analysis in TNT v1.1^61^. We first conducted a ‘new technology’ search (with default parameters for sectorial search, ratchet, tree drift, and tree fusion), which recovered a minimum length tree in 10 replicates. This procedure aims to broadly sample tree space and identify individual tree islands. We then subjected the recovered most parsimonious trees (MPTs) to a traditional search with TBR branch swapping, which more fully explores the tree islands found in the ‘new technology’ search. This process returned a total of 33,104 MPTs of 566 steps (consistency index = 0.484, retention index = 0.676). Bremer values were used to assess clade support.

## Additional Information

**How to cite this article**: Lü, J. *et al*. A Late Cretaceous diversification of Asian oviraptorid dinosaurs: evidence from a new species preserved in an unusual posture. *Sci. Rep.* **6**, 35780; doi: 10.1038/srep35780 (2016).

**Publisher’s note**: Springer Nature remains neutral with regard to jurisdictional claims in published maps and institutional affiliations.

## Supplementary Material

Supplementary Information

## Figures and Tables

**Figure 1:**
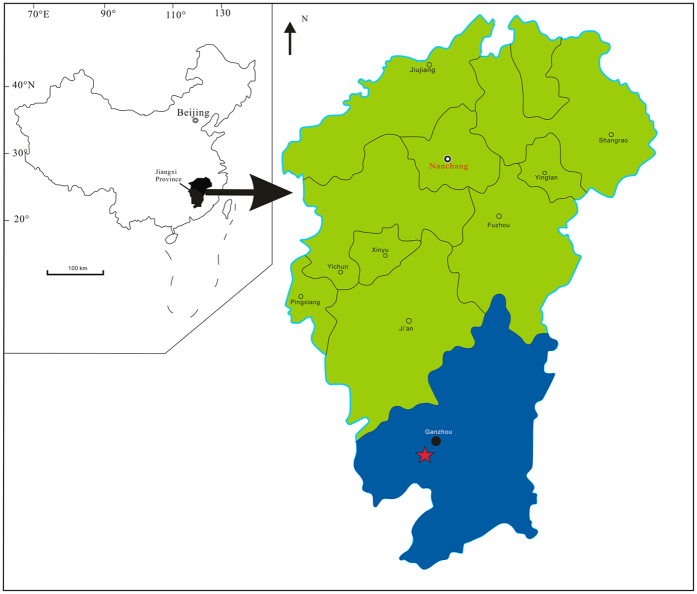
Map of the fossil locality near Ganzhou, Jiangxi Province, southern China. The solid five-pointed star represents the fossil site. Modified from Lü *et al*.^29^.
