# Supplementary material for: A Late Cretaceous diversification of Asian oviraptorid dinosaurs: evidence from a new species preserved in an unusual posture
Source: Sci Rep. 2016 Nov 10;6:35780. doi: 10.1038/srep35780 (PMC5103654; doi:10.1038/srep35780)
Supplement: Supplementary Information [file srep35780-s1.pdf]

Supplementary information

A Late Cretaceous diversification of Asian oviraptorid dinosaurs: evidence from a new species preserved in an unusual posture

Junchang Lü[1*], Rongjun Chen[2], Stephen L. Brusatte[3], Yangxiao Zhu[2], and Caizhi Shen[1]

Contents

1. Geological information about the fossil site of ***Tongtianlong limosus*** gen. et sp. nov.
2. Measurements of holotype of ***Tongtianlong limosus*** gen. et sp. nov.
3. Fig. S2. The skull of ***Tongtianlong limosus*** gen. et sp. nov.
4. Fig. S3. The photo of the left pes of ***Tongtianlong limosus*** gen. et sp. nov.
5. Character list and datamatrix for phylogenetic analysis
6. References

1. Geological information about the site where *Tongtianlong limosus* gen. et sp. nov. was discovered

The specimen was non-professionally collected by a local farmer. The first author (JL) later visited the fossil site with the farmer who collected the specimen. According to the GPS data crossed referenced with geological maps, the site is a part of the Nanxiong Formation[S1]. The formation is dominated by purple-reddish sandstones, fine sandstones and mudstones, and is regarded as late Late Cretaceous in age [S1], although future geological and stratigraphic work on this formation is clearly needed to confirm this. The rocks at the site, and the rocks entombing the specimen, are characteristic of the lithologies of the Nanxiong Formation described above (Fig.S1).

The late Late Cretaceous (Maastrichtian) age of the site is based on the following chain of inference. The *Tongtianlong*-bearing strata in the Ganzhou Basin are regarded as belonging to the Nanxiong Formation by the Chinese Bureau of Geology and Mineral Resources of Jiangxi Province [S1]. The type section of the Nanxiong Formation is in the nearby Nanxiong Basin, and the Nanxiong Formation has been identified in the Ganzhou Basin based on regional correlations. These correlations are based on lithology and vertebrate biostratigraphy, namely the discovery of the turtle *Nanxiongchelys*[S1] and the same types of dinosaur eggs in both areas[S2]. Many dinosaur eggs have been found in close proximity to *Tongtianlong*, in the same facies. These are not only similar to oviraptorid eggs found in the Nanxiong Basin, but also similar to eggs excavated from the Nanxiong Formation of the Hongcheng Basin, also near the city of Ganzhou[S2]. The eggs provide a key source of evidence linking together the lithologically similar units in the various basins as all belonging to the Nanxiong Formation.

The age of the Nanxiong Formation in the Nanxiong Basin has been the subject of

extensive research, and a wealth of biostratigraphic, radioisotopic, and paleomagnetic data support a very latest Cretaceous (Maastrichtian age) [S3]. Most important is the radioisotopic dating of three basalt flows 1000 m below the last dinosaur occurrences in the formation [S4]. The whole-rock $^{40}Ar/^{39}Ar$ dates indicate a maximum age of 66.7 ± 0.3 Ma, which is squarely in the Maastrichtian.

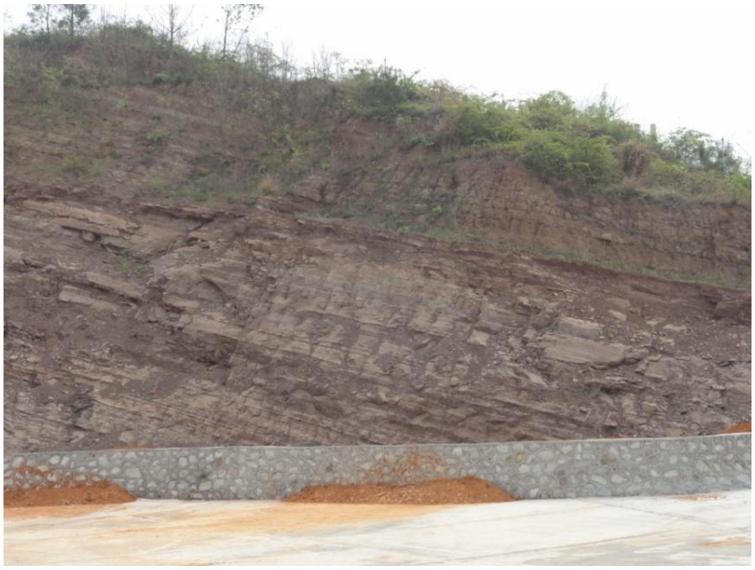

Fig. S1. The fossil site where ***Tongtianlong limosus*** **gen. et sp. nov.** was discovered.

2. Table S1. Measurements (cm) of the holotype of *Tongtianlong limosus* gen. et sp. nov.

	Length	Width	Height
Skull	14.5 (preserved); 18.9(estimated) (from anterior tip to the posterior margin of the skull)	10 (between articular ends of the quadrates)	10 (top of the skull to the junction of jugal with the postorbital)
Preorbital skull	8.3	-	-
Premaxilla	2.7		
Maxilla	3.6	-	-
Orbit	4.1		4
Antorbital fenenstra	2.8	1.4	
External nasal opening	2.5	1.4	
Nasal	8		
Frontal	1.5		
Parietal	2		
Supratemporal fenestra	3	1.5	
Lateral temporal fenestra	2		4
Lower jaw	11.9 (anterior tip to the distal end of the retroarticular process)	5.5	4
Dentary	5 (anterior tip to the end of the posterodorsal process)		3.1
Mandibular symphysis	2.3 (measured along its sagittal midline)	-	-

Mandibular fenestra	2.5	-	2.3
Cervical vertebrae 1-13	41 (total measurement of neck)		
2nd	1.7 (centrum)	1.6 (centrum); 3.6 (between lateral margins of the postzygapophyses)	
3rd	3.5 (between the anterior margin of the prezygapophysis to the posterior margin of the postzygapophysis)	3.5 (between lateral margins of the prezygapophyses); 4 (between the lateral margins of the postzygapophysis)	
4th	3.9 (between the anterior margin of the prezygapophysis to the posterior margin of the postzygapophysis)	4 (between lateral margins of the prezygapophyses); 4.5 (between the lateral margins of the postzygapophysis)	
5th	4(between the anterior margin of the prezygapophysis to the posterior margin of the postzygapophysis)	4(between lateral margins of the prezygapophyses); 4.4 (between the lateral margins of the postzygapophysis)	
6th	4.5 (between the anterior margin of the prezygapophysis to the posterior margin of the postzygapophysis)	4 (between lateral margins of the prezygapophyses); 4 (between the lateral margins of the postzygapophysis)	
7th	4.5 (between the anterior margin of the prezygapophysis to the posterior margin of the	4(between lateral margins of the prezygapophyses); 3.8 (between the lateral margins of the postzygapophysis)	

	postzygapophysis)		
8th	4.5	-;-	
9th	-	-; 3.6(between the lateral margins of the postzygapophysis)	
Dorsal vertebrae	35		
Sacral vertebrae			
Caudal vertebrae			
Scapula	18.5	1.8	
Coracoid	7.2		
Humerus	13.1	5 (proximal end); 3 (distal end); 1.5 (shaft)	
Deltopectoral crest of humerus	6.4	1.2	
Radius	10.2	1 (shaft)	
Femur	25		
Tibia	32		4.4 (distal end)
Metatarsals II-V	12.5;13.5;11;3.8	-	

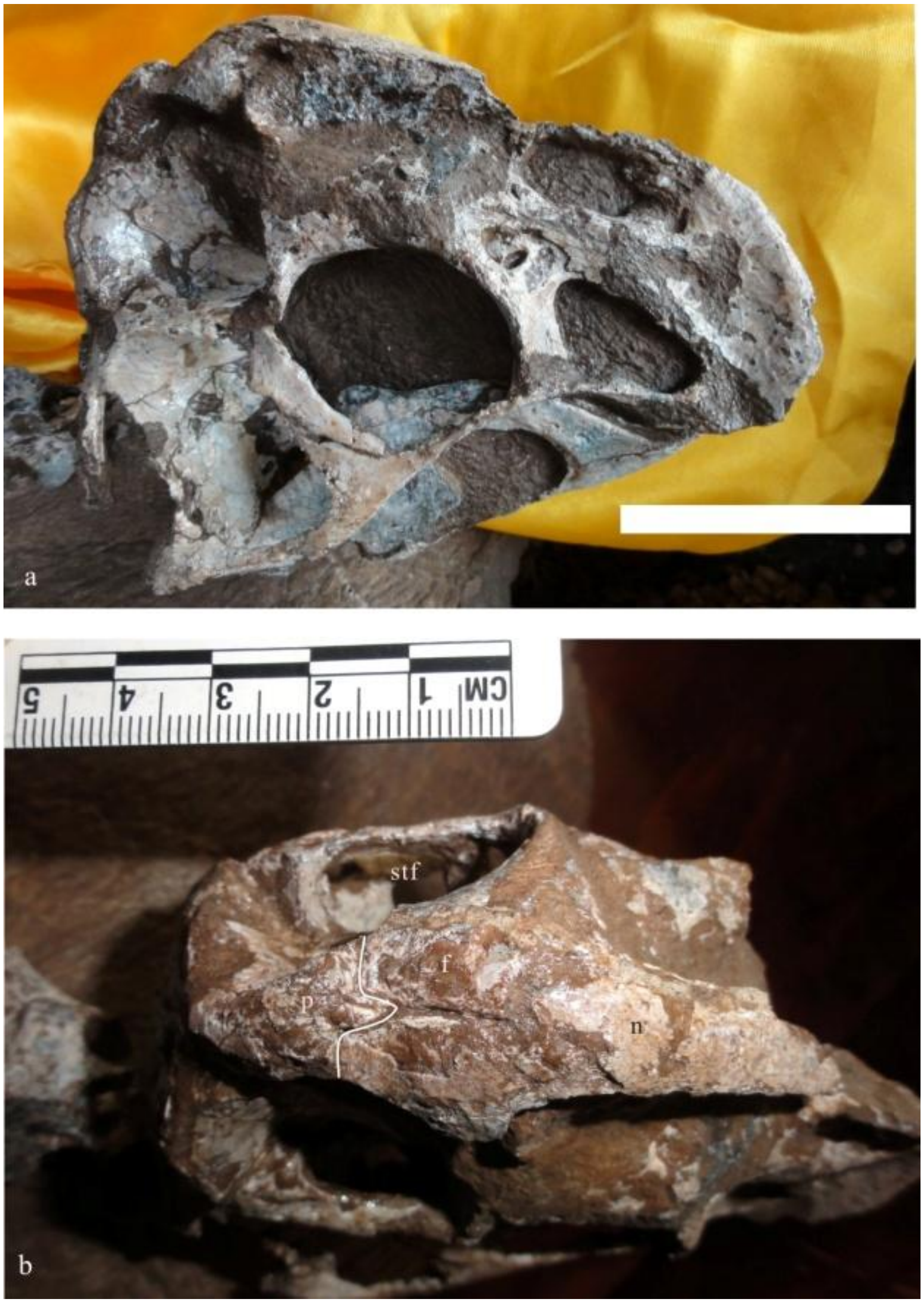

3. Fig. S2. The skull and lower jaw of ***Tongtianlong limosus*** gen. et sp. nov. in lateral view (a), showing the structure of the dentary, and in dorsal view (b), showing the structure of the parietal. Scale bar is 5 cm in a. Abbreviations: f, frontal; n, nasal; p, parietal; stf, supratemporal fenestra.

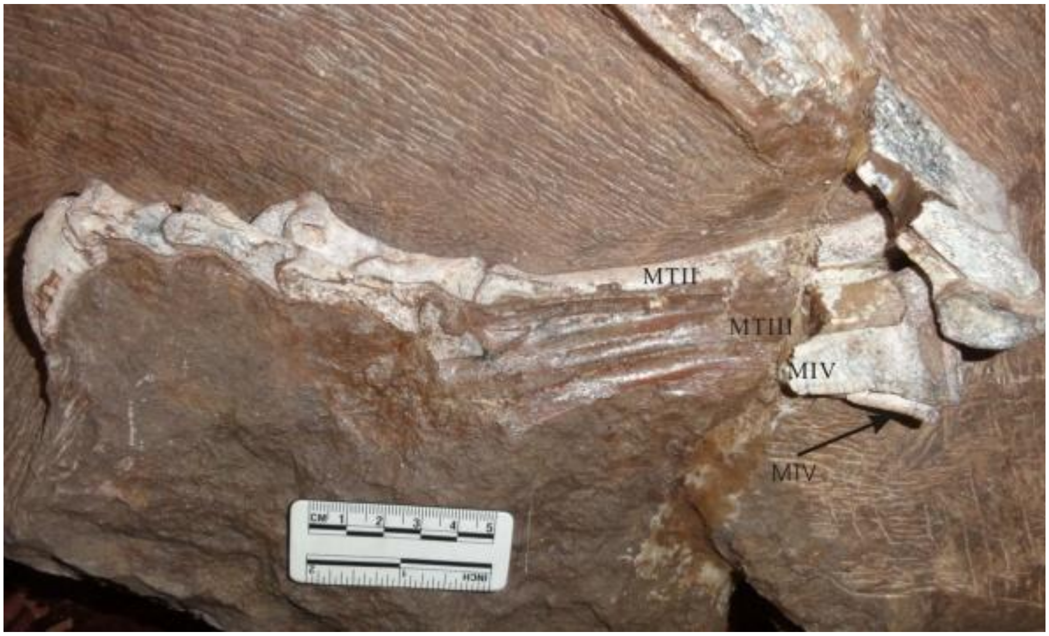

4. Fig. S3. The photo of the left pes of ***Tongtianlong limosus*** gen. et sp. nov. Abbreviations: MTII –MTV, metatarsals II to V

5. Character list and datamatrix for phylogenetic analysis:

5.1 Character list (from Lü et al.[S5], based on that of Lamanna et al.[S6]. We have slightly modified the character list relative to previous publications, to take into account some of the recommendations for scoring anatomical characters presented by Brazeau[S6]. Most importantly, we have divided various characters that were previously multistate (including absence) into multiple characters denoting presence/absence and morphological differences. Changes relative to previous analyses are indicated by yellow highlight.

1. Ratio of the preorbital skull length to the basal skull length: 0.6 or more (0); less than 0.6 (1) (Lü et al., 2013b).

2. Pneumatized crest-like prominence on the skull roof: absent (0); present (1).

3. Ratio of the width (across premaxilla–maxilla suture) of the snout to its length: less than 0.3 (0); 0.3–0.4 (1); more than 0.4 (2) (Lü et al., 2013b). (ORDERED)

4. Ratio of the length of the tomial margin of the premaxilla to the premaxilla height (ventral to the external naris): 0.7 or less (0); 1.0–1.4 (1); more than 1.7 (2). (ORDERED)

5. Inclination of the anteroventral margin of the premaxilla relative to the horizontally positioned ventral margin of the jugal: vertical (0); posterodorsal (1); anterodorsal (2).

6. Ventral projection of the premaxilla below the ventral margin of the maxilla: absent (0); present (1). (modified).

7. Ventral projection of the premaxilla below the ventral margin of the maxilla: small (0); significant (1) (new added).

8. Share of the premaxilla (ventral) in the basal skull length: 0.10 or less (0); 0.12 or more

(1).

9. Pneumatization of the premaxilla: absent (0); present (1).

10. Ratio of the length of the maxilla (in lateral view) to the basal skull length: 0.4–0.7 (0); less than 0.4 (1) (Lü et al., 2013b).

11. Subantorbital portion of the maxilla: not inset medially (0); inset medially (1).

12. Palatal shelf of the maxilla with two longitudinal ridges and a tooth-like ventral process: absent (0); present (1).

13. Ventral margins of maxilla and jugal: margins form a straight line (0); the ventral margin of the maxilla slopes anteroventrally, its longitudinal axis at an angle of ca. 120° to the longitudinal axis of the jugal (1).

14. Rim around antorbital fossa: well pronounced (0); poorly delimited (1).

15. Antorbital fossa: bordered anteriorly by the maxilla (0); bordered anteriorly by the premaxilla (1).

16. Accessory maxillary fenestrae: absent (0); at least one accessory fenestra present (1).

17. Nasal along midline: longer than frontal (0); shorter than or as long as the frontal (1).

18. Nasals: separate (0); fused (1).

19. Subnarial process of the nasal: long (0); short (1).

20. Shape of the narial opening: longitudinally oval (0); teardrop-shaped, slightly longer than wide (1); much longer than wide (2). (ORDERED).

21. Nasal recesses: absent (0); present (1).

22. External naris position relative to the antorbital fossa: naris and fossa widely separated (0); posterior margin of the naris reaching the fossa (1); overlapping anterodorsally most of the fossa (2). (ORDERED)

23. Ventral margin of the external naris: at the level of the maxilla (0); dorsal to the maxilla (1).

24. Prefrontal: present (0); absent or fused with the lacrimal (1).

25. Lacrimal shaft: not projecting outward beyond the orbital plane and lateral surface of the snout (0); the middle part of the shaft projecting laterally to form a flattened transverse bar in front of the eye (1).

26. Lacrimal recesses: absent (0); present (1).

27. Ratio of the length of the orbit to the length of the antorbital fossa: 0.7–0.9 (0); 1.2 or more (1).

28. Ratio of the length of the parietal to the length of the frontal: 0.6 or less (0); 1.0 or more (1).

29. Pneumatization of skull roof bones: absent (0); present (1).

30. Sagittal crest along the interparietal contact: absent (0); present (1).

31. Supratemporal fossa: invading the frontal (0); not invading the frontal (1).

32.Infratemporal fenestra: dorsoventrally elongate, narrow anteroposteriorly (0); subquadrate, its anteroposterior length comparable to the orbital length (1).

33. Pneumatization of the squamosal: absent (0); present (1).

34. Cotyle-like incision on the ventrolateral margin of the squamosal (for reception of the dorsal end of the ascending process of the quadratojugal): absent (0); present (1).

35. Ventral ramus of the jugal: deep dorsoventrally and flattened mediolaterally (0); shallow dorsoventrally or rod-shaped (1).

36. Jugal process of the postorbital: not extending ventrally below two-thirds of the orbit height (0); long, extending ventrally close to the base of the postorbital process of the jugal

(1).

37. Postorbital process of the jugal: posterodorsally inclined (0); perpendicular to the ventral ramus of the jugal (1). (modified)

38. Postorbital process of the jugal: present (0); absent (1) (new added)

39. Jugal–postorbital contact: present (0); absent (1).

40. Quadratojugal process of the jugal in lateral view: forked (0); not forked (1); fused with the quadratojugal (2).

41. Quadratojugal–squamosal contact: absent (0); present (1).

42. Ascending (squamosal) process of the quadratojugal: bordering ca. the ventral half, or less, of the infratemporal fenestra (0); bordering the ventral two-thirds or more of the infratemporal fenestra (1) (modified).

43. Ascending (squamosal) process of the quadratojugal: present (0); absent (1). (new added).

44. Angle between the ascending and jugal processes of the quadratojugal: ca. 90 ° (0); less than 90 ° (1).

45. Quadrate process of the quadratojugal: well developed, extending posteriorly or posteroventrally beyond the posterior margin of the ascending process (0); not extending beyond the posterior margin of the ascending process (1).

46. Dorsal part of the quadrate: erect (0); bent backward (1).

47. Otic process of the quadrate: articulating only with the squamosal (0); articulating with the squamosal and the lateral wall of the braincase (1).

48. Pneumatization of the quadrate: absent (0); present (1).

49. Lateral accessory process on the distal end of the quadrate for articulation with the quadratojugal: absent (0); present (1).

50. Lateral cotyle for the quadratojugal on the quadrate: absent (0); present (1).

51. Mandibular condyles of quadrate: posterior to the occipital condyle (0); in the same vertical plane as the occipital condyle (1); anterior to the occipital condyle (2). (ORDERED)

52. Nuchal transverse crest: pronounced (0); not pronounced (1).

53. Occiput position in relation to the ventral margin of the jugal–quadratojugal bar: about perpendicular (0); inclined anterodorsally (1).

54. Paroccipital process: directed laterally (0); directed ventrally (1).

55. Foramen magnum: smaller than or equal in size to the occipital condyle (0); larger than the occipital condyle (1).

56. Basal tubera: modestly pronounced (0); well developed, widely separated (1).

57. Pneumatization of the basisphenoid: weak or absent (0); extensive (1).

58. Basipterygoid processes: well developed (0); strongly reduced (1) (modified).

59. Basipterygoid processes:present (0); absent (1) (new added).

60. Parasphenoid rostrum: horizontal or anterodorsally directed (0); sloping anteroventrally (1).

61. Depression in the periotic region: absent (0); present (1).

62. Pneumatization of the periotic region: absent or weak (0); extensive (1).

63. Quadrate ramus of the pterygoid: distant from the braincase wall (0); overlapping the braincase (1).

64. Pterygoid basal process for contact with the basisphenoid: absent (0); present (1).

65. Ectopterygoid position: lateral to the pterygoid (0); anterior to the pterygoid (1).

66. Ectopterygoid contacts with the maxilla and lacrimal: absent (0); present (1).

67. Ectopterygoid: short anteroposteriorly with a hook-like jugal process (0); elongate, shaped

like a Viking ship, without a hook-like process (1).

68. Massive pterygoid–ectopterygoid longitudinal bar: absent (0); present (1).

69. Palate extending below the cheek margin: absent (0); present (1).

70. Palatine: tetraradiate or trapezoidal (0); triradiate, without a jugal process (1); developed in horizontal, longitudinal, and transverse planes perpendicular to each other (2).

71. Pterygoid wing of the palatine: dorsal to the pterygoid (0); ventral to the pterygoid (1).

72. Maxillary process of the palatine: shorter than the vomeral process (0); longer than the vomeral process (1).

73. Vomer: distant from the parasphenoid rostrum (0); approaching or in contact with the parasphenoid rostrum (1).

74. Suborbital (ectopterygoid–palatine) fenestra: well developed (0); closed or reduced (1).

75. Jaw joint: distant from the midline of the skull (0); close to the skull midline (1).

76. Movable intramandibular joint: present (0); suppressed (1).

77. Mandibular symphysis: loose (0); tightly sutured (1); fused (2). (ORDERED).

78. Extended symphyseal shelf at the mandibular symphysis: absent (0); present (1).

79. Downturned symphyseal portion of the dentary: absent (0); present (1).

80. U-shaped mandibular symphysis: absent (0); present (1).

81. Ratio of the length of the retroarticular process to the total mandibular length: less than 0.05 or the process absent (0); ca. 0.10 (1).

82. Dentary: elongate (0); proportionately short and deep, with maximum depth of dentary between 25% and 50% of dentary length (with length measured from the tip of the jaw to the end of the posterodorsal process) (1); extremely short and deep, with maximum depth 50% or more of dentary length (2) (ORDERED) (Longrich et al., 2013).

83. Ratio of the height of the external mandibular fenestra to the length of the fenestra: 0.2–0.5 (0); 0.7–1.0 (1). (modified).

84. External mandibular fenestra: present (0); absent (1). (new added).

85. Ratio of the length of the external mandibular fenestra to total mandibular length: absent or not more than 0.10 (0); between 0.15 and 0.20 (1), greater than 0.25 (2). (ORDERED).

86. Process of the surangular dividing the external mandibular fenestra: absent (0); short and broad (1); elongate and spike-like (2) (ORDERED) (Longrich et al., 2010).

87. Coossification of the articular with the surangular: absent (0); present (1).

88. Mandibular rami in dorsal view: straight (0); laterally bowed at midlength (1).

89. Anterodorsal margin of dentary in lateral view: straight (0); concave (1); broadly concave (2) (ORDERED) (Longrich et al., 2013).

90. Posterior margin of the dentary: incised, producing two posterior processes (0); oblique (1).

91. Posterodorsal process of the dentary long and shallow: present (0); absent (1).

92. Posteroventral process of the dentary shallow and long, extending posteriorly at least to the posterior border of the external mandibular fenestra: absent (0); present (1).

93. Coronoid process: posteriorly positioned and vertically projected (0); anteriorly positioned, near the midpoint of the jaw, with a medially hooked apex (1) (Longrich et al., 2013).

94. Surangular foramen: present (0); absent (1).

95. Mandibular articular facet for the quadrate: comprising the surangular and the articular (0); formed exclusively of the articular (1).

96. Mandibular articular facet for the quadrate: with one or two cotyles (0); convex in lateral

view, transversely wide (1).

97. Position of the quadrate articular surface relative to the level of the adjoining dorsal margin of the mandibular ramus: ventral (0); moderately elevated, quadrate articulation grades smoothly into remainder of mandible (1); highly elevated, anterior and posterior margins of quadrate articulation at nearly right angles to remainder of mandible (2) (ORDERED) (Lamanna et al. 2014).

98. Anterior part of the prearticular: deep, approaching the dorsal margin of the mandible (0); shallow, strap-like, not approaching the dorsal mandibular margin (1).

99. Splenial: subtriangular, approaching the dorsal mandibular margin (0); strap-like, shallow, not approaching the margin (1).

100. Mandibular adductor fossa: anteriorly delimited, occupying the posterior part of the mandible (0); large, anteriorly and dorsally extended, not delimited anteriorly (1).

101. Coronoid bone: well developed (0); strongly reduced (1). (modified).

102. Coronoid bone: present (0); absent (1). (new added).

103. Premaxillary teeth: present (0); absent (1).

104. Maxillary tooth row: extends at least to the level of the preorbital bar (0); does not reach the level of the preorbital bar (1); maxillary teeth absent (2). (ORDERED)

105. Dentary teeth: present (0); absent from tip of jaw but present posteriorly (1); absent (2) (ORDERED) (Longrich et al., 2013).

106. Number of cervicals (excluding cervicodorsal): not more than 10 (0); more than 10 (1).

107. Anterior articular facets of the centra in the anterior postaxial cervicals: not inclined or only slightly inclined (0); strongly inclined posteroventrally, almost continuous with the ventral surfaces of the centra (1).

108. Centra of the anterior cervicals: not extending posteriorly beyond their respective neural arches (0); extending posteriorly beyond their respective neural arches (1).

109. Epipophyses on the postaxial cervicals: in the form of a low crest or rugosity (0); prong-shaped (1).

110. Cervical ribs in adults: loosely attached to vertebrae (0); firmly attached (1); fused (2). (ORDERED).

111. Shafts of cervical ribs: longer than their respective centra (0); not longer than their respective centra (1).

112. Lateral pneumatic fossae (‘pleurocoels’) on the dorsal centra: absent (0); present (1).

113. Ossified uncinate processes on the dorsal ribs: absent (0); present (1).

114. Number of vertebrae included in the synsacrum in adults: not more than 5 (0); 6 (1); 7–8 (2). (ORDERED).

115. Sacral spines in adults: unfused (0); fused (1).

116. Lateral pneumatic fossae on the sacral centra: absent (0); present (1).

117. Transition point on the caudals: absent (0); present (1).

118. Number of caudals with transverse processes: 15 or more (0); fewer than 15 (1).

119. Lateral pneumatic fossae on the caudal centra: absent (0); present at least in the anterior part of the tail (1).

120. Neural spines confined to: at least 23 anterior caudals (0); at most 16 anterior caudals (1).

121. Number of caudals: more than 35 (0); 30 or fewer (1).

122. Posterior caudal prezygapophyses: overlapping less than half of the centrum of the preceding vertebra (0); overlapping at least half of the centrum of the preceding vertebra (1).

123 Hypapophyses in the cervicodorsal vertebral region: absent (0); small (1); prominent (2). (ORDERED)

124. Posterior hemal arches: deeper than long (0); longer than deep (1).

125. Ratio of the length of the scapula to the length of the humerus: 0.7 or less (0); 0.8-1.1 (1), 1.2 or more (2). (ORDERED).

126. Acromion: projecting dorsally (0); projecting anteriorly (1); everted laterally (2).

127. Posteroventral process of the coracoid: absent or short, not extending beyond the glenoid diameter (0); long, posteroventrally extending beyond the glenoid (1).

128. Orientation of the glenoid on the pectoral girdle: posteroventral (0); lateral (1).

129. Deltopectoral crest: low, its width equal to, or smaller than, the shaft diameter (0); expanded, wider than the shaft diameter (1).

130. Extent of the deltopectoral crest (measured from the humeral head to the apex of the crest): about the proximal third of the humerus length or less (0); ca. 40%–50% of the humerus length (1).

131. Shaft of the ulna: straight (0); bowed, convex posteriorly (1).

132. Ratio of the length of the radius to the length of the humerus: 0.80 or less (0); 0.85 or more (1).

133. Combined lengths of manual phalanges III-1 and III-2: greater than the length of phalanx III-3 (0); less than or equal to the length of phalanx III-3 (1).

134. Ratio of the length of metacarpal I to the length of metacarpal II: 0.5 or more (0); less than 0.5 (1).

135. Proximal margin of metacarpal I in dorsal view: straight, horizontal (0); angled due to a medial extent of the carpal trochlea (1).

136. Metacarpal II relative to metacarpal III: shorter (0); subequal (1); longer (2). (ORDERED).

137. Ratio of the length of metacarpal II to the length of the humerus: 0.4 or less (0); more than 0.4 (1).

138. Ratio of the length of the manus to the length of the humerus plus the radius: less than 0.50 (0), between 0.50 and 0.65 (1), greater than 0.65 (2). (ORDERED).

139. Ratio of the length of the manus to the length of the femur: 0.3–0.6 (0); more than 0.7 (1).

140. Ratio of the length of the humerus to the length of the femur: 0.50–0.69 (0); 0.70 or more (1) (Lü et al., 2013b).

141. Dorsal margins of opposite iliac blades: well separated from each other (0); close to or contacting each other along their medial sections (1).

142. Dorsal margin of the ilium along the central portion of the blade: straight (0); arched (1); concave (2) [modified based on *Nomingia*]

143. Preacetabular process of the ilium relative to the postacetabular process (lengths measured from the center of the acetabulum): shorter or equal (0); longer (1).

144. Preacetabular process: not expanded or weakly expanded ventrally below the level of the dorsal acetabular margin (0); expanded ventrally well below the level of the dorsal acetabular margin (1).

145. Morphology of the ventral margin of the preacetabular process: cuppedicus fossa absent, margin transversely narrow (0); cuppedicus fossa or a wide shelf present (1); margin flat, wide at least close to the pubic peduncle (2).

146. Anteroventral extension of the preacetabular process: absent (0); present (1). (modified).

147. Anteroventral extension of the preacetabular process: with rounded tip (0); hook-like (1). (new added).

148. Posterior end of the postacetabular process: truncated or broadly rounded (0); narrowed or acuminate (1).

149. Anteroposterior length of the pubic peduncle: about the same as that of the ischial peduncle (0); distinctly greater than that of the ischial peduncle (1).

150. Dorsoventral extension of the pubic peduncle: level with the ischial peduncle (0); deeper than the ischial peduncle (1).

151. Ratio of the length of the ilium to the length of the femur: 0.50–0.79 (0); 0.80 or more (1) (Lü et al., 2013b).

152. Pelvis: propubic (0); mesopubic (1); opisthopubic (2). (ORDERED).

153. Pubic shaft: straight (0); concave anteriorly (1).

154. Pubic foot: anterior process absent or shorter than posterior process (0); two processes equally long (1); anterior process longer than posterior process (2). (ORDERED).

155. Posterior margin of the ischial shaft: straight or almost straight (0); distinctly concave (1).

156. Greater trochanter of the femur: weakly separated, or not separated, from the femoral head (0); distinctly separated from the femoral head (1).

157. Anterior and greater trochanters: separated (0); contacting (1).

158. Dorsal extremity of the anterior trochanter: well below the greater trochanter (0); about level with the greater trochanter (1).

159. Fourth trochanter: well developed (0); weakly developed or absent (1).

160. Adductor fossa and the associated anteromedial crest on the distal femur: weak or absent

(0); well developed (1).

161. Distal projection of the fibular condyle of the femur beyond the tibial condyle: absent (0); present (1).

162. Ascending process of the astragalus: as tall as wide across the base (0); taller than wide (1).

163. Distal tarsals: not fused with the metatarsus (0); fused with the metatarsus (1).

164. Proximal coossification of metatarsals II–IV: absent (0); present (1).

165. Arctometatarsus: absent (0); present, but only proximalmost extreme of metatarsal III obscured from anterior view in articulated metatarsus (1); present, proximal ~half of metatarsal III obscured from anterior view in articulated metatarsus (2) (Lamanna et al., 2014). (ORDERED).

166. Length of metatarsal I constituting: more than 50% of metatarsal II length (0); less than 50% of metatarsal II length (1); metatarsal I absent (2). (ORDERED).

167. Ratio of the maximum length of the metatarsus to the length of the femur: less than 0.3 (0), between 0.4 and 0.6 (1), 0.7-0.8 (2). (ORDERED).

168. Crenulated tomial margin of the premaxilla: absent (0); present (1).

169. Frontals: flat or weakly arched, not strongly projecting above orbit in lateral view (0); strongly arched, projecting well above orbit in lateral view to contribute to nasal–frontal crest (1).

170. Exoccipital: short, weakly projecting (0); strongly projects ventrally beyond squamosal in lateral view, approaching ventral end of quadrate (1).

171. Dentary posterodorsal ramus: straight or weakly curved (0); strongly bowed dorsally (1).

172. Dentary symphyseal ventral process: absent (0); prominent process present on posteroventral surface of symphysis (1).

173. Dentary anteroventral margin in lateral view: straight or weakly downturned (0); strongly downturned (1).

174. Lateral surface of dentary: smooth (0); bearing a deep fossa, sometimes with associated pneumatopore (1).

175. Angular: contributes extensively to the border of the external mandibular fenestra (0); largely excluded by surangular (1).

176. Surangular with an anteroposteriorly elongate flange on the ventral edge: absent (0); present (1).

177. External mandibular fenestra: elongate (0); height subequal to length (1).

178. Dentary contribution to external mandibular fenestra relative to length of dentary: no more than 50% (0); exceeds 50% (1).

179. Metacarpal I expanded ventrally to cover ventral surface of metacarpal II: absent (0); present (1).

180. Unguals of manual digits II and III: strongly curved (0); weakly curved (1).

181. Manual phalanx I-1: slender (0); more robust than II-1 (1); more than 200% diameter of II-1 (2) (ORDERED).

182. Manual phalanx III-3: longer than phalanx III-2 (0); shorter than or equal in length to III-2 (1).

183. Manual phalanx II-2: longer than II-1 (0); subequal to or slightly shorter than II-1 (1); distinctly shorter than II-1 (2) (ORDERED) (Longrich et al., 2013).

184. Manual digit II: elongate, with combined lengths of manual phalanges II-1 and II-2

longer than metacarpal II (0); combined lengths of manual phalanges II-1 and II-2 subequal to metacarpal II (1) (Lamanna et al., 2014).

185. Ischium strongly bent posteriorly at midshaft, distal end forms an angle of at least 60 º with proximal end: absent (0); present (1).

186. Metatarsus: elongate (0); short, length does not exceed 300% of proximal width (1).

187. Ilium: tall (0); low and anteroposteriorly elongate, height less than 25% of length (1).

188. Anterior blade of ilium shallower than posterior blade: absent (0); present (1).

189. External naris: placed anteriorly (0); extends posteriorly, with posterior end lying above antorbital fenestra (1).

190. Premaxillae, nasal processes anteroposteriorly expanded and mediolaterally compressed to form a bladelike internarial bar: absent (0); present (1).

191. Dentary, anterodorsal tip of beak: projecting upwards (0); projecting anterodorsally, tip of beak projecting at an angle of 45 ° or less relative to the ventral margin of the symphysis (1).

192. Dentary symphysis with interior surface bearing vascular grooves and associated foramina: absent (0); present (1).

193. Dentary symphysis bearing an hourglass-shaped ventral depression: absent (0); present (1).

194. Meckelian groove terminates: on the inside of the dentary (0); on the ventral surface of the symphysis (1).

195. Lingual triturating shelf: absent (0); present (1).

196. Symphyseal ridges inside the tip of the beak: absent (0); present but weakly developed (1); present and well developed (2) (ORDERED).

197. Lingual ridges inside the lateral occlusal surface of beak: absent (0); present (1).

198. Posteroventral process of dentary: straight (0); bowed ventrally (1).

199. Dentaries pneumatized: absent (0); present (1).

200. Dentary: participates in dorsal border of the external mandibular fenestra (0); excluded by anterior extension of the surangular (1).

201. Dentary: participates in ventral border of external mandibular fenestra (0); excluded by anterior extension of the angular (1).

202. Surangular and angular divided by posterior extension of the external mandibular fenestra: absent (0); present (1).

203. Posterior end of the surangular: deep (0); shallow, subequal to or shallower than angular (1).

204. Surangular: deep anteriorly (0); strap-like (1).

205. Retroarticular process extends: posteriorly (0); posteroventrally (1).

206. Metacarpal I: proportionately broad (0); long and slender, diameter 20% of length (1).

207. Manual phalanx I-1: longer than II-2 (0); subequal to II-2 (1); shorter than II-2 (2) (ORDERED).

208. Ischiadic peduncle of pubis with prominent medial fossa: absent (0); present (?).

209. Ischium, obturator process located: distally (0); at midshaft of ischium (1).

210. Anterior margin of obturator process: straight or convex (0); distinctly concave (1).

211. Accessory trochanter of femur: weakly developed (0); prominent, subrectangular flange or finger-like process (1).

212. Metatarsal III: with an ovoid or subtriangular cross section (0); anteroposteriorly flattened, with a concave posterior surface (1).

213. Paroccipital process: elongate and slender, with dorsal and ventral edges nearly parallel (0); short and deep with convex distal end (1).

214. Mandibular articulation surface: as long as ventral end of quadrate (0); twice or more as long as quadrate surface, allowing anteroposterior movement of mandible (1).

215. Scars for interspinous ligaments in dorsal vertebrae terminate: at apex of neural spine (0); ventral to apex of neural spine (1)

216. Sternum, distinct lateral xiphoid process posterior to costal margin: absent (0); present (1)

217. Anterior edge of sternum: grooved for reception of coracoids (0); without grooves (1)

218. Deltopectoral crest: large and distinct, proximal end of humerus quadrangular in anterior view (0); less pronounced, forming an arc rather than being quadrangular (1).

219. Ischium: more than two-thirds of pubis length (0); two-thirds or less of pubis length (1)

220. Lateral ridge of femur: absent or represented only by faint rugosity (0); distinctly raised from shaft, mound-like (1)

221. Surangular, distinct groove on dorsal surface: present (0); absent (1).

222. Vomer, position: level with other palatal elements (0); ventral to other palatal elements (1)

223. Calcaneum: excludes astragalus from reaching lateral margin of tarsus (0); small process of astragalus protrudes through a circular opening in edge of calcaneum to reach lateral margin of tarsus (1).

224. Depression on lateral surface of dentary immediately anterior to external mandibular fenestra: absent (0); present (1).

225. Groove on ventrolateral edge of angular to receive posteroventral branch of dentary:

absent (0); present (1).

226. Posteroventral branch of dentary twisted so that lateral surface of branch faces somewhat ventrally: absent (0); present (1).

227. Premaxilla, large, presumably pneumatic foramen at anteroventral corner of narial fossa: absent (0); present (1).

228. Accessory opening at anterodorsal extreme of snout: absent (0); present (1).

229. Development of symphyseal shelf of mandible: limited, anteroposterior length of mandibular symphysis (as measured on midline) less than 20% total anteroposterior length of mandible (0); intermediate, length of symphysis greater than 20% but less than 25% length of mandible (1); extensive, length of symphysis greater than 25% mandibular length (2) (ORDERED).

230. Prominent flange or shelf arising from lateral surface of dentary: absent (0); present (1).

231. Base of retroarticular process: considerably wider mediolaterally than tall dorsoventrally (0); approximately as wide as tall (1); considerably taller than wide (2). (ORDERED).

232. Posteriormost caudal vertebrae fused, forming a pygostyle-like structure: absent (0); present (1).

233. Humeral shaft: straight or nearly straight (0); strongly bowed laterally (1).

234. Proximodorsal extensor ‘lip’ on manual unguals: weak (i.e., continuous or nearly continuous with remainder of dorsal surface of ungual) and/or absent (0); prominent (‘set off’ from remainder of dorsal surface by distinct change in slope immediately distal to ‘lip’) (1).

235. Pubic process of ischium, ‘hooked’ anterodistal extension: absent (0); present (1).

236. Distal margin of obturator process: straight (0); distinctly concave, apex of obturator process angled distally (1).

237. Proximolateral edge of metatarsal IV attenuated into pointed process: absent (0); present (1).

5.2 Datamatrix (in TNT form)

xread
237 43
Herrerasaurus_ischigualastensis
000000-0000000000000000000000000000000000000000000000000000000000000000
00000000000000000000000000000000010?000000000100?0000101??000000000000-00001
0000000000000000000?000?0000110010100??000??0000101??00??0000???00?1000?00000
0?00000
Velociraptor_mongoliensis
000100-0000001010010100101000100000000000100000000010000100000000000000000
*0000000000100001100000000000010010110111101101011101-110101111011100001101111201*0
01110001001100000000?0000000011000?0000??0010000100000000110011?000?00000000
0000
Archaeopteryx_lithographica
000?10-0000001011010011000100 01?1000-1110??0?1??0020????000????0000001?1???
1000010-11000011-01000000-10100??0101?00?1101100121110011111210110 11010-111020101
?0100?1101200?0000?0?0?00000001000?0000??001?000000?010010?0?111??00?00000?000
0?0
Incisivosaurus_gauthieri
100110-101000011?0111210011?01?00?00000?100010?0??200111?10?10?01??111?0101?
2101110020??00011??11?1110011???
?????????????00000000100??????????0010000000000000???????1???????00???01000???
????
Caudipteryx_zoui
10?110-?01??0?1110?0?21?01?10???0110000111001 00?????????????????????0????????
0??1111??2???0001??????11??0220?????010????0?1??10???1011?1020100?0012110110?02
1100???1000121??0???0??0?00?001000?000?00000000?01000?0010???0101????0?00000?0
00000
Avimimus_portentosus
????????????0???????????????10110??1?-1121-101??00?101111?10????????????????0
?1??111???01?????11000???-11?21000110?110??0???2?02100111?1?????00000?1110?1002
0101001111222100010000?0???????00??00?00????0??0000??0??10110??11110????????0?0
?000
Microvenator_celer
???
??1111?2?????11001?????????????2???0??1??????????0????1?1011??1????0???1111?11?11
2???11101????????0101???001[0
1]?????00??0000000?0???????0?1??1???0??1?0???1?????0??00???
Oviraptor_philoceratops

1????????????1?11?????21?????????1??10000????0??????????????????????????1?????
????1?121022??10011???1??1???22???0??1?????????????1???101101?211??????????????
????????????????0???001001000000??0??1????????????????0??????????0??11?0?0???0
??00???
Rinchenia_mongoliensis
11122??0111110111???1211111111?111101001110001111111?11111-11?11111112111111111
2102?0110011111111-11221110011?1010010102?12101000??????01101111101???111111?
?????1101?001011????????00111??????00000001????????1?10???1110?0?0?0???????
Citipati_osmolskae
1122211111110110111121111111111111100111011?1??11111??101111?1111121?111111111
21022011001111111110122???????1????????????????0?101?2?????????????????????????
???????11011001011?01010?0??110000000000000000100?????010100?01110?001000100?0?
Zamyn_Khondt_oviraptorid
1122211111110111111211111111111111001110111111111111-1111111111211111111111210
2201100111111111-11221110111?101001010210210101101121110100121010011121111110001
01101?001011?0000010??110?0000000000?0100???00?1?100??11???001?0??00??0
Khaan_mckennai
10221101?1111011?112121111111111?110000111000?111?111111???0??1?????1??????111
11121021011001111111???1221110??1????00?0102??210??1101021110?0001100001?121111
1?11000101001100100100101001111110?000?001000001000000?01?1100?1?10101000?0000[0
1]0
Conchoraptor_gracilis
1012110111111011111212111111111111110000111100?1111111?1111-11111111112111111111111?
102?01100111111111211221110111?2010010101112101111001211101000210000111211111111100
010101?????????110???1??11?????????????????0??????11?00?011?0?0000011?0??0
Machairasaurus_leptonychus
??
??????????????????????????????2????????????????????????????0012????????????????
??????????????????????????111110??????????????????????00????????????????????????
???1???
Nemegtomaia_barsboldi
1112010111111011111121011111111?110000101010 11?111111?1???11?111111121111111111
2102?11100111111?11-11221110[0
2]11?201?????????????????????????10002???00?1????11?????????10111101011?12?11??1
1110000000010000001000??0??1???1??11?1?1?0001?000??
Heyuannia_huangi
???????????????????????????????????1???
????1??21021??1001????1????????21???21-12????1?????121?11000002?20???01?1010011
1?1????????????????1?1?1011112?2101?1?????????0?00000100?0?0?0101?00?1?11?0???0
?00001?
Ingenia_yanshini
????????1???????????????11?????1?1100001110?0111111???111???11?1??111?1???111
11112102?01100111111111-1122?110011?20100101010121011000012020010012100001112 1111
111100010?011?1010111121210111110000000000000000100?0000?1?100001?1??0???0?000010
Gigantoraptor_erlianensis
??

11211112002011100111112?????1?2????????????0010?0??00?00000?11?1??1????????????1
??1111111000?0???0001010000?????????0???0001001110100???0??????1?????110??112?
10???
Caenagnathasia_martinsoni
??
??21?1?1?????12????????????????2???
??????????????????0001???0?????????????0111111?1??????????????????????????????0
???????
Elmisaurus_elegans
??
??
??????????1[0
1]1?????????????????????0???????????????????????????1?????????????????????????1
Leptorhynchos_gaddisi
??
??21?1?[1 2]?????1[1
2]???????????????2??101
??????0???????????????????0111121?1??????????????????????????????????????1
Chirostenotes_pergracilis
??
???????????????????????????????????????111??????????10?????0????1??11??111101?
??11??1?1100110?????????????000101000?????????????????12?1111??????????????????
???1000
Caenagnathus_collinsi
??
1121011100201120011111111-1??2???
????????????????00010100?????????????1101121011111l1????????????????1??010??201??
????
Anzu_wyliei
?1??10-1100?1100???????????????????0?0000???????1011??100?10??1??0?011?????1
121011?0020112001111111?1-11221110211?1?10010?02?02100010????1110???1?11?1?110211
111111?????1?000010100000??01?0??0?1011211011111??1111?110101101?00101021201110
?
Hagryphus_giganteus
??
??0010?????????????????
???????????????????????????000010???????????????????????2????????????????????????
???1???
Elmisaurus_rarus
??
??001??????????????????
??????????101??????????????000010?0????????????????????11???10??????????????????
???1??1
Nomingia_gobiensis
??
???????????????????????????????????00?1?01?00101020????????????????1110110011111

011111111???????????????????????1?00????????????????????111???0???1???1????????1
??10?
Epichirostenotes_curriei
??????????01?010??????1??????????????????????????????110?10?1??0??????????
????????????????????????????????????0?1?111??1???2?????????????????????????????????
021??????????????????????????????1?????????????????????111??0?0???1???????????
????10?
Alberta_dentary_morph_3
??
?12111?1?????1200?????????????2??
??????????????????0001???0????????????0111121?10???????????????????????0?????0
???????
Caenagnathus_sternbergi
??
1121111100201120011112?11-2???2??
?????????????????00010100????????????111112111111111????????????????1??010???0??
?????
Macrophalangia_canadensis
??
???
??????????0011????????????????????0??
??????0
Banji_long
11?210-111???0111102121111111011??111001?????0??11?1111?????????1111121?0111??
11?21021??000110??1?11??122?1??
???????????11111001011??????????110??????0?00000????????????????00?0?000?0????
???
Caudipteryx_dongi
??
???????????????????????????????????????01?0?????????????0010?1?20100?011?1101100
0211?????100012?????????????00?001000?????????????????01?10?????0??1???????????
??00000
Ganzhousaurus_nankangensis
??
?1[0 1]111?210?[1
2]011001?????111????2?????????????0???0??
0001????1?001?11???????1????????0?001?10?0???????0????????????111???0???????
Jiangxisaurus_ganzhouensis
1??????????1?????????????11???????1?0001????????112?????1-1?????1?01121??01
1111112102111100111111?????122??10??11??1??1?????121?1110?0120????0?????1???????
????????????1??110010111?2??????????111??2?0100000100??????1?1?0?????0011?00??0
0???
Nankangia_jiangxiensis
??
?11101?2??????100?????????????2???????1???1??1??0?0011001?????????01110110111111
211001111????????1101??0???????1?00??11?????0?1???1????11???????01???11?0???0??

0?00?
Ojoraptorsaurus_boerei
??
???
02???1?????????????????????
???????
Shixinggia_oblita
??
?????????????????????????????????????1?2?1??1?????????????????????110000-000??
???1????1??????????????????????????01??
???????
Similicaudipteryx_yixianensis
??
???????????????????????????????????0?11?0?1?0??1?2????????1????????0?001?111110?
????????0??0?12????????????????????00???????????????????????0??????????????????
?10????
Wulatelong_gobiensis
11?22101?11?1?1???02121?111?1??1?1110001???00??11????????????????1111??????
??????????101????1??11?????12????????1???100????2???????????1??????1100?111111112
1???????000?01????????????0??????011????????????000????1????1?10?101???????????
?0??0
Yulong_mini
102010-111011011011112101011101 0?0100001110010?111110110???0????????0?????111
1111210220110011[0 1]1111?1-2122?100??1????001?10??[0
1]??0000101?21010?110?100??0??0?1?0??11?001010?11001011?11000?100110??????0?000
0010???????1??????1110?01000??00???
Huanansaurus_ganzhouensis
11222111111101101?11211111111111?110001110111?11100111?????1???11?11????11111
112102201100111111?????122?110211????????????????001101?211????????????????????
?????????11011000011?01000????1?1?0????0100000110?????01???0??1??011??101?01??
?
Tongtianlong
10222??11111101?0112121111110111110001100001111?21110?????011?????1?????1111
011210210110011011??????1221110201??01?01??0?01?1001?0???????01??????????1121??
????1000?011010001001??????11??111??????01000001???10?101?010??1?100010001?0??
00;
ccode

/1-[0	*/1-[1*	*/1+[2*	*/1+[3*	*/1-[4*	*/1-[5*
/1-[6	*/1-[7*	*/1-[8*	*/1-[9*	*/1-[10*	*/1-[11*
/1-[12	*/1-[13*	*/1-[14*	*/1-[15*	*/1-[16*	*/1-[17*
/1-[18	*/1+[19*	*/1-[20*	*/1+[21*	*/1-[22*	*/1-[23*
/1-[24	*/1-[25*	*/1-[26*	*/1-[27*	*/1-[28*	*/1-[29*
/1-[30	*/1-[31*	*/1-[32*	*/1-[33*	*/1-[34*	*/1-[35*
/1-[36	*/1-[37*	*/1-[38*	*/1-[39*	*/1-[40*	*/1-[41*
/1-[42	*/1-[43*	*/1-[44*	*/1-[45*	*/1-[46*	*/1-[47*
/1-[48	*/1-[49*	*/1+[50*	*/1-[51*	*/1-[52*	*/1-[53*

/1-[54	*/1-[55*	*/1-[56*	*/1-[57*	*/1-[58*	*/1-[59*
/1-[60	*/1-[61*	*/1-[62*	*/1-[63*	*/1-[64*	*/1-[65*
/1-[66	*/1-[67*	*/1-[68*	*/1-[69*	*/1-[70*	*/1-[71*
/1-[72	*/1-[73*	*/1-[74*	*/1-[75*	*/1+[76*	*/1-[77*
/1-[78	*/1-[79*	*/1-[80*	*/1+[81*	*/1-[82*	*/1-[83*
/1+[84	*/1+[85*	*/1-[86*	*/1-[87*	*/1+[88*	*/1-[89*
/1-[90	*/1-[91*	*/1-[92*	*/1-[93*	*/1-[94*	*/1-[95*
/1+[96	*/1-[97*	*/1-[98*	*/1-[99*	*/1-[100*	*/1-[101*
/1-[102	*/1+[103*	*/1+[104*	*/1-[105*	*/1-[106*	*/1-[107*
/1-[108	*/1+[109*	*/1-[110*	*/1-[111*	*/1-[112*	*/1+[113*
/1-[114	*/1-[115*	*/1-[116*	*/1-[117*	*/1-[118*	*/1-[119*
/1-[120	*/1-[121*	*/1+[122*	*/1-[123*	*/1+[124*	*/1-[125*
/1-[126	*/1-[127*	*/1-[128*	*/1-[129*	*/1-[130*	*/1-[131*
/1-[132	*/1-[133*	*/1-[134*	*/1+[135*	*/1-[136*	*/1+[137*
/1-[138	*/1-[139*	*/1-[140*	*/1-[141*	*/1-[142*	*/1-[143*
/1-[144	*/1-[145*	*/1-[146*	*/1-[147*	*/1-[148*	*/1-[149*
/1-[150	*/1+[151*	*/1-[152*	*/1+[153*	*/1-[154*	*/1-[155*
/1-[156	*/1-[157*	*/1-[158*	*/1-[159*	*/1-[160*	*/1-[161*
/1-[162	*/1-[163*	*/1+[164*	*/1+[165*	*/1+[166*	*/1-[167*
/1-[168	*/1-[169*	*/1-[170*	*/1-[171*	*/1-[172*	*/1-[173*
/1-[174	*/1-[175*	*/1-[176*	*/1-[177*	*/1-[178*	*/1-[179*
/1+[180	*/1-[181*	*/1+[182*	*/1-[183*	*/1-[184*	*/1-[185*
/1-[186	*/1-[187*	*/1-[188*	*/1-[189*	*/1-[190*	*/1-[191*
/1-[192	*/1-[193*	*/1-[194*	*/1+[195*	*/1-[196*	*/1-[197*
/1-[198	*/1-[199*	*/1-[200*	*/1-[201*	*/1-[202*	*/1-[203*
/1-[204	*/1-[205*	*/1+[206*	*/1-[207*	*/1-[208*	*/1-[209*
/1-[210	*/1-[211*	*/1-[212*	*/1-[213*	*/1-[214*	*/1-[215*
/1-[216	*/1-[217*	*/1-[218*	*/1-[219*	*/1-[220*	*/1-[221*
/1-[222	*/1-[223*	*/1-[224*	*/1-[225*	*/1-[226*	*/1-[227*
/1+[228	*/1-[229*	*/1+[230*	*/1-[231*	*/1-[232*	*/1-[233*
/1-[234	*/1-[235*	*/1-[236*	*/1-[237;*		

;

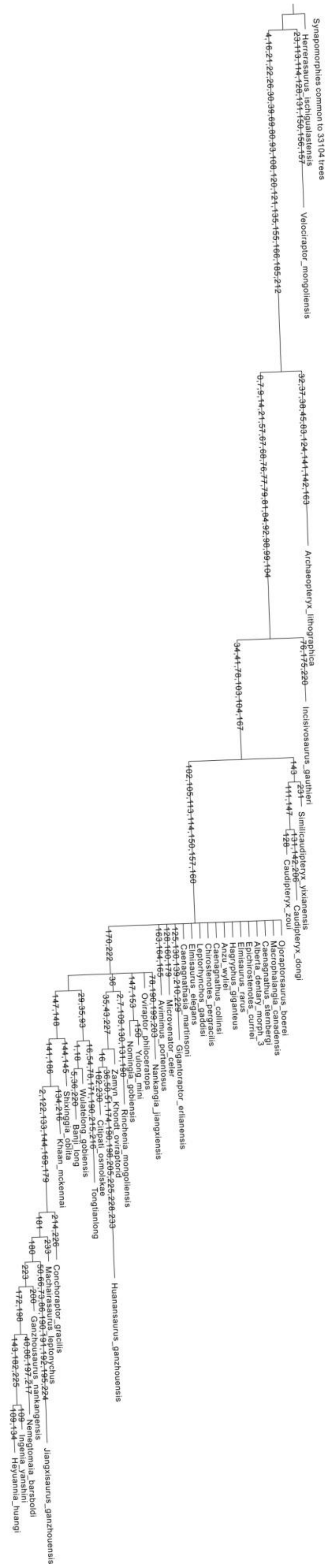
Synapomorphies common to 33104 trees
Herrerasaurus_ischigualastensis
Velociraptor_mongoliensis
Archaeopteryx_lithographica
Incisivosaurus_gauthieri
Similicaudipteryx_yixianensis
Caudipteryx_zoui
Caudipteryx_dongi
Ojoraptorsaurus_boerei
Macrophalangia_canadensis
Caenagnathus_sternbergi
Alberta_dentary_morph_3
Epichirostenotes_curriei
Elmisaurus_rarus
Hagryphus_giganteus
Anzu_wyliei
Caenagnathus_collinsi
Chirostenotes_pergracilis
Leptorhynchos_gaddisi
Elmisaurus_elegans
Caenagnathasia_martinsoni
Gigantoraptor_erlianensis
Microvenator_celer
Avimimus_portentosus
Nankangia_jiangxiensis
Oviraptor_philoceratops
Yulong_mini
Nomingia_gobiensis
Rinchenia_mongoliensis
Zamyn_Khondt_oviraptorid
Citipati_osmolskae
Tongtianlong
Wulatelong_gobiensis
Banji_long
Shixinggia_oblita
Khaan_mckennai
Huanansaurus_ganzhouensis
Conchoraptor_gracilis
Machairasaurus_leptonychus
Ganzhousaurus_nankangensis
Nemegtomaia_barsboldi
Ingenia_yanshini
Heyuannia_huangi
Jiangxisaurus_ganzhouensis

Fig.S4. The strict consensus of 33104 most parsimonious trees with synapomorphies

6. References

S1. Bureau of Geology and Mineral Resources of Jiangxi Province. *Regional Geology of Jiangxi Province*, 921p. (Geological Publishing House, Beijing) (1984).

S2. Sato, T., Cheng, Y.N., Wu, X.C., Zelenitsky, D. K., & Hsiao, Y.F. A pair of shelled eggs Inside a female dinosaur. *Science*, 308:375 (2005).

S3. Taylor, L. H., Jacobs, L. L. & Downs, W. R. A review of the Cretaceous-Paleogene boundary in the Nanxiong Basin. *Papers from the 2005 Heyuan International Dinosaur Symposium*, p. 39-59 (2006).

S4. Rigby, J. K. Jr., Snee, L. W., Unruh, D. M., Harland, S. S., Guan, J., Li, F., Rigby, J. K. Sr. & Kowalis, B. J. $^{40}Ar/^{39}Ar$ and U-Pb dates for dinosaur extinction, Nanxiong Basin, Guangdong Province, People's Republic of China. Geological Society of America Abstracts with Program 25: A296 (1993).

S5. Lü, J.C. et al. A new oviraptorid Dinosaur (Dinosauria: Oviraptorosauria) from the Late Cretaceous of Southern China and its paleobiogeographical implications. *Sci. Rep.* **5,** 11490; doi: 10.1038/srep11490 (2015).

S6. Lamanna, M. C., Sues, H. D., Schachner, E. R. & Lyson, T. R. A new large-bodied oviraptorosaurian theropod dinosaur from the Latest Cretaceous of Western North America. *PLoS ONE* **9,** e92022 (2014).

S7. Brazeau, M D. Problematic character coding methods in morphology and their effects. *Biological Journal of the Linnean Society*, 104: 489–498 (2011).
